# Dermal features derived from optoacoustic tomograms via machine learning correlate microangiopathy phenotypes with diabetes stage

**DOI:** 10.1038/s41551-023-01151-w

**Published:** 2023-12-04

**Authors:** Angelos Karlas, Nikoletta Katsouli, Nikolina-Alexia Fasoula, Michail Bariotakis, Nikolaos-Kosmas Chlis, Murad Omar, Hailong He, Dimitrios Iakovakis, Christoph Schäffer, Michael Kallmayer, Martin Füchtenbusch, Annette Ziegler, Hans-Henning Eckstein, Leontios Hadjileontiadis, Vasilis Ntziachristos

**Affiliations:** 1 Institute of Biological and Medical Imaging, Helmholtz Zentrum München, Neuherberg, Germany; 2 Chair of Biological Imaging at the Central Institute for Translational Cancer Research (TranslaTUM), School of Medicine, Technical University of Munich, Munich, Germany; 3 Department for Vascular and Endovascular Surgery, Klinikum rechts der Isar, Technical University of Munich (TUM), Munich, Germany; 4 DZHK (German Centre for Cardiovascular Research), partner site Munich Heart Alliance, Munich, Germany; 5 Institute of Computational Biology, Helmholtz Zentrum München, Neuherberg, Germany; 6 Department of Biomedical Engineering, Healthcare Engineering Innovation Center (HEIC), Khalifa University, Abu Dhabi, United Arab Emirates; 7 Department of Electrical and Computer Engineering, Aristotle University of Thessaloniki, Thessaloniki, Greece; 8 Diabetes Center at Marienplatz, Munich, Germany; 9 Forschergruppe Diabetes e.V., Helmholtz Zentrum München, Neuherberg, Germany; 10 Institute of Diabetes Research, Helmholtz Zentrum München, Neuherberg, Germany; 11 Forschergruppe Diabetes, Klinikum rechts der Isar, Technical University of Munich (TUM), Munich, Germany; 12 Munich Institute of Robotics and Machine Intelligence (MIRMI), Technical University of Munich, Munich, Germany

**Keywords:** Biomarkers, Diabetes complications

## Abstract

Skin microangiopathy has been associated with diabetes. Here we show that skin-microangiopathy phenotypes in humans can be correlated with diabetes stage via morphophysiological cutaneous features extracted from raster-scan optoacoustic mesoscopy (RSOM) images of skin on the leg. We obtained 199 RSOM images from 115 participants (40 healthy and 75 with diabetes), and used machine learning to segment skin layers and microvasculature to identify clinically explainable features pertaining to different depths and scales of detail that provided the highest predictive power. Features in the dermal layer at the scale of detail of 0.1–1 mm (such as the number of junction-to-junction branches) were highly sensitive to diabetes stage. A ‘microangiopathy score’ compiling the 32 most-relevant features predicted the presence of diabetes with an area under the receiver operating characteristic curve of 0.84. The analysis of morphophysiological cutaneous features via RSOM may allow for the discovery of diabetes biomarkers in the skin and for the monitoring of diabetes status.

## Abstract (web summary)

Skin-microangiopathy phenotypes can be correlated with diabetes stage by leveraging clinically explainable morphophysiological features obtained from the analysis, via machine learning, of raster-scan optoacoustic mesoscopy images of skin on the leg.

## Main

Diabetes affects tissue microvasculature and causes microangiopathy, a condition that distorts the function of many organs, including the human skin^1–4^. Recent work has shown that skin microangiopathy occurs early in the course of diabetes and that it may precede microvascular complications in other organs^5^ as well as macrovascular complications and overt hyperglycaemia (≥200 mg dl^−1^ blood-glucose level), the hallmark of diabetes^6,7^. However, insights into diabetes effects as a function of diabetes progression and stage are less known. Scarce histopathological studies have observed the thickening of the basement membrane, a decrease of the capillary lumen and microvascular density, or a reduction in the dimensions of the capillaries at conditions associated with later stages of the disease, such as foot ulcerations^8–10^. However, histology is not well suited for disseminated disease owing to the invasive nature of the required skin biopsy. Moreover, the histological process may alter the morphology of the samples because of the processes of biopsy extraction, tissue processing into slices, loss of blood pressure and other changes in the physiological characteristics from the in vivo tissue^11^.

Therefore, microvascular changes are not customarily studied in clinical research and clinical care, and there is little information available on how diabetes complications are manifested in terms of alterations of the cutaneous microvasculature. Nevertheless, the study and possibly the routine examination of the skin microvasculature could contain valuable information for understanding disease progression and offering quantitative metrics for disease staging. Quantitative description of systemic changes owing to disease progression could enhance the assessment of diabetes progression in current clinical practice, which is typically based on crude symptoms such the appearance of neuropathy, kidney malfunction or cardiovascular problems^12–14^.

**Fig. 1:**
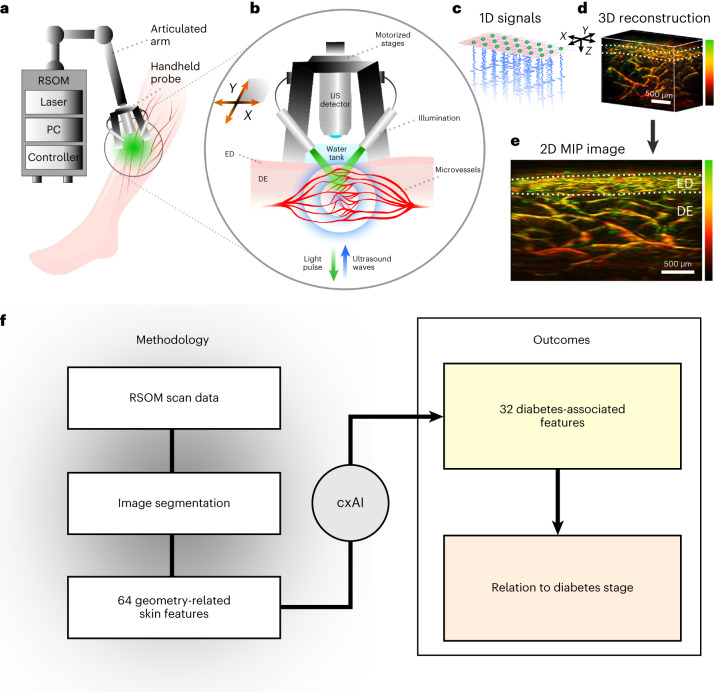
Study workflow. **a**, RSOM scanning setup. The controller regulates the motion of the stages. **b**, RSOM principle of operation. The handheld probe with mounted illumination is fixed on the skin and the motorized stages move the ultrasound detector along the *XY* plane over the scanned skin region. For each light pulse, a number of ultrasound waves are produced. ED, epidermal layer; DE, dermal layer. **c**, The ultrasound detector records an ultrasound 1D signal at each scanning point in a single-pulse-per-signal manner. **d**, Recorded ultrasound signals are reconstructed into a volumetric 3D RSOM image. The different colours represent different frequencies (green, high frequencies; red, low frequencies). The white dotted lines represent the limits of the epidermal/SVP layer. **e**, Finally, a tomographic 2D MIP (along the *Y* axis) RSOM image is produced. Scale bars, 500 μm. **f**, After segmenting the microvasculature in the RSOM images, we extracted 64 geometry-related skin features. Next, a cxAI approach identified the 32 most diabetes-associated features which were further related to diabetes stage/progression.

Among methods that can non-invasively visualize microvasculature in vivo, optoacoustic mesoscopy has emerged as an imaging modality providing the most detailed imaging because of the strong contrast it obtains from vascular structures, its ability to offer high-resolution three-dimensional (3D) visualization through the entire epidermal and dermal layers, and because it can reach depths of up to several millimetres depending on the wavelength employed (Fig. 1)^15,16^. In particular, raster-scan optoacoustic mesoscopy (RSOM) has assessed diabetes-related changes in the dermal microvasculature^17^, whereas ultra-wideband RSOM has been applied to assess microvascular and inflammatory markers of skin disease through the entire dermal layer at resolutions in the 7–30 μm range (Fig. 1), providing information not accessible by any other non-invasive method today^18–21^. Hence, RSOM offers the opportunity to study the relation of skin features to diabetes complications. Although growing interest in the area has recently shown the application of smartphone-based photoplethysmography for the non-invasive detection of diabetes^22^, the diagnosis of diabetes can be reliably and more accurately achieved with a blood test. Moreover, photoplethysmography only offers two-dimensional (2D) surface-weighted observations on bulk skin features that do not contain the wealth of information offered by RSOM, in particular the ability to visualize 3D microvasculature phenotypes at resolutions of a few micrometres to a few tens of micrometres.

**Fig. 2:**
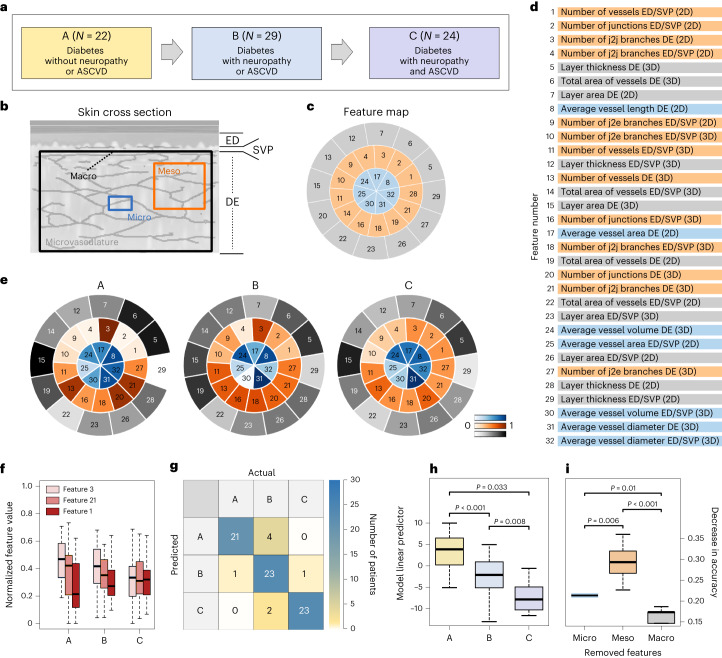
Diabetes progression analysis and multiscale interpretation of the features. **a**, Schematic representation of the 3 groups of patients with diabetes (A, B and C) that represent increasing diabetes stage/progression. **b**, Schematic representation of the skin cross-section with the different defined scales of microvascular detail, that is, macroscale, mesoscale and microscale. **c**, Segmental map of the 32 selected features employed in further analysis. **d**, The 32 finally selected diabetes-relevant features. Different colours correspond to different scales of detail: grey, macroscale features; orange, mesoscale features; blue, microscale features. Feature numbers correspond to their relative importance/relevance to diabetes, as described in Fig. 3. j2j, junction-to-junction; j2e, junction-to-endpoint. **e**, Comparison of the mean feature segmental maps among the 3 groups of diabetes stage (A, B and C) in all scales of detail. The intensity of each segment corresponds to the normalized mean value of the feature for each group, as described by the three colour bars (blue, orange, grey). **f**, The 3 skin features that showed the highest change across A, B and C. Features 3 (number of j2j branches in dermal layer – 2D) and 21 (number of j2j branches in dermal layer – 3D) decrease and feature 1 (number of vessels in epidermal/SVP layer – 2D) increases. The normalization of the features was performed for every group (A, B, C) separately against the maximum value on a per-feature basis. The *P* values of all changes are given in Table 1. **g**, Confusion matrix of the ordinal classification model for the 3 diabetes progression groups A, B and C. **h**, Linear predictor of the diabetes progression model across the 3 progression groups. **i**, Decrease in accuracy of the disease progression classification model caused by the removal of each scale of features. The removal of the mesoscale features leads to the highest decrease in accuracy, showcasing that this scale of detail is the most important for monitoring diabetes severity/progression. All *P* values were calculated using a two-sided Student’s *t*-test. For the boxplots, the centre line represents the median, the box limits the first and third quartiles, and the whiskers (minima and maxima) 1.5× the interquartile range.

**Fig. 3:**
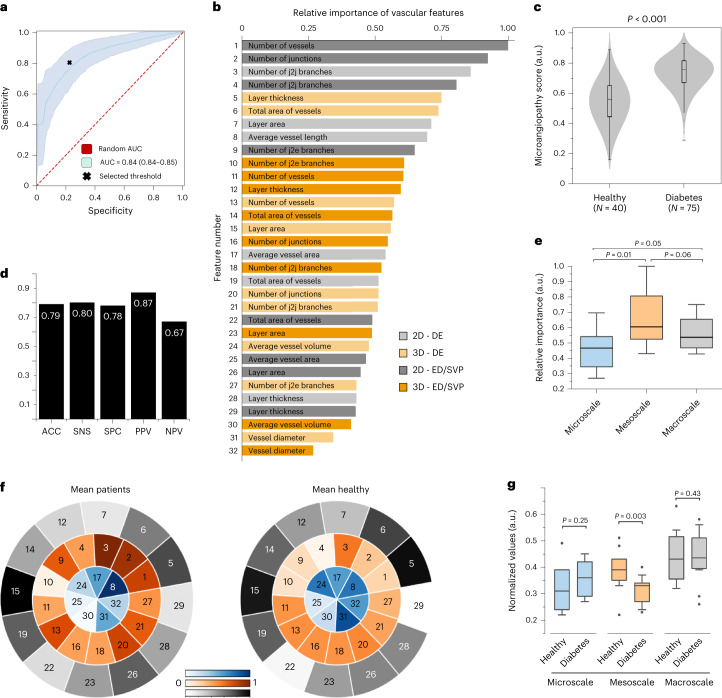
Importance of vascular features, classification and subgroup analysis for the microangiopathy score. **a**, ROC curve of the developed classification/diabetes detection scheme with the selected threshold (0.65) of the microangiopathy score. The blue area indicates the 95% CI of ROC curves for multiple iterations. **b**, Relative importance of the 32 selected 2D and 3D RSOM-extracted vascular features for the calculation of the microangiopathy score. **c**, Classification of healthy volunteers and patients with diabetes based on the calculated microangiopathy score. **d**, Efficacy metrics of the proposed method for diabetes detection. **e**, Statistical description of the groups of macroscale, mesoscale and microscale vascular features based on their relative importance for diabetes detection by means of the microangiopathy score. **f**, Comparison of the mean feature segmental maps among the healthy and diabetes groups in all scales of detail. The intensity of each segment corresponds to the normalized mean value of the feature for each group, as described by the three colour bars (blue, orange, grey). **g**, Statistical comparison of the normalized values, against the maximum value on a per-feature basis, of the selected features between healthy volunteers and patients for all three scales of detail. All *P* values were calculated using a two-sided Student’s *t*-test. For the boxplots, the centre line represents the median, the box limits the first and third quartiles, and the whiskers (minima and maxima) 1.5× the interquartile ranges.

In this work, we apply a clinically explainable AI (cxAI) approach (Fig. 1) to 3D RSOM images from the unperturbed skin at resolutions reaching single-capillary dimensions to relate skin features to diabetes complications, thereby facilitating clinical interpretability^23,24^. We obtained RSOM images from 115 participants (40 healthy and 75 patients with diagnosed diabetes) and automatically segmented the skin layers and microvasculature with machine learning via U-NETs. The participants represented a cross-section of patients with type-I or type-II diabetes since in this introductory pilot study we aimed at establishing evidence of the global implications of diabetes stage on the skin microvasculature. We hope that this study stimulates the investigation of more targeted questions relating to the relationships between skin microangiopathy and specific diabetes groups or diabetes indicators.

Termed cxAI, the methodology developed here processes RSOM-extracted features that have clinical relevance, as they describe structural markers of microanatomy, as opposed to other potential RSOM-extracted features that could be computed but have no clinical meaning (for example, the skewness or the derivative of an RSOM signal). Using geometric formulas to analyse the segmented skin layers and microvasculature, we quantified 64 such explainable features that could relate to the underlying pathophysiological processes of diabetes. A second AI step (random-forest classifier) identified 32 of these features that were most related to diabetes, that is, the features that achieved the best classification performance between healthy volunteers and patients with diabetes (see online Methods). These features were subsequently categorized into three scales of architectural detail, that is, the microscale (<100 μm), the mesoscale (≈100–1,000 μm) and the macroscale (>1,000 μm). We found that previously unobserved dermal features are prominently affected by diabetes, often in ways that differ from changes in more superficial skin layers. This observation explains why methods that only capture bulk signals from the skin, and thereby only an average of the disease’s effects in different layers and size scales, may lead to inconclusive observations. We further identify that features that belong to the mesoscale, which describes the morphology and organization of the microvascular network, are more sensitive to diabetes and diabetes severity compared with microscale and macroscale features. The association of the 32 selected features with diabetes progression was performed against clinically assessed diabetes grade on the basis of symptoms, such as the presence of peripheral neuropathy or atherosclerotic cardiovascular disease (ASCVD). Finally, we compiled the 32 most diabetes-relevant skin features into a ‘microangiopathy score’ and showed that the score can be used to discriminate diabetes with an area under a receiver operating characteristic (ROC) curve (AUC) of 0.84 (and with sensitivity (SNS)/specificity (SPC) of 0.80/0.78). The predictive value of the microangiopathy score is controlled against age variations.

The implications of our approach are: (1) knowledge regarding diabetes stage, as using the abilities of cxAI, we build knowledge on the skin microvasculature features that are globally most sensitive to diabetes stage (Table 1) from a list of 32 explainable features; (2), exploratory ability, as cxAI/RSOM enables the interrogation of diabetes effects on the (micro-)vasculature (Fig. 2), an exploration that sheds light on the unperturbed skin. We identified that many statistically significant features are located in the dermal layer (which so far has not been studied in detail by any other non-invasive method); (3) dissemination potential, because with the skin being easily accessible, the optoacoustic approach carries dissemination potential to population studies that relate diabetes stage to skin features in unperturbed environments in vivo. (4) quantification and diabetes score, as the study provides preliminary evidence that cxAI/RSOM can leverage the skin as a window to determine quantitative metrics of the effects of diabetes on the microvasculature. These metrics may be integrated into diabetes indices to offer personalized and higher-precision characterization of diabetes status or of the effects of intervention on each patient; and (5) future applicability, as the understanding of skin microangiopathy may impact the staging and monitoring of diabetes progression^25^.

**Table 1:** Description of the 7 features that change monotonically (decrease/increase) with diabetes stage/progression and *P* values of changes

Feature number	Feature description	Feature scale	Feature change	*P* values		
				A vs B	B vs C	A vs C
3	Number of junction-to-junction branches in the dermal layer (2D)	Mesoscale	**↓**	0.047*	0.153	0.01**
5	Thickness of the dermal layer (3D)	Macroscale	**↓**	0.464	0.188	0.252
6	Total area of vessels in the dermal layer (3D)	Macroscale	**↓**	0.317	0.326	0.477
8	Average vessel length in the dermal layer (2D)	Microscale	**↓**	0.156	0.336	0.315
13	Number of vessels in the dermal layer (3D)	Mesoscale	**↓**	0.395	0.364	0.276
20	Number of junctions in the dermal layer (3D)	Mesoscale	**↓**	0.387	0.373	0.276
21	Number of junction-to-junction branches in the dermal layer (3D)	Mesoscale	**↓**	0.349	0.221	0.331
1	Number of vessels in the epidermal/SVP layer (2D)	Mesoscale	**↑**	0.257	0.386	0.323
2	Number of junctions in the epidermal/SVP layer (2D)	Mesoscale	**↑**	0.263	0.444	0.377
4	Number of junction-to-junction branches in the epidermal/SVP layer (2D)	Mesoscale	**↑**	0.447	0.266	0.377
25	Average vessel area in the epidermal/SVP layer (2D)	Microscale	**↑**	0.109	0.354	0.09*

*P < 0.05, **P ≤ 0.01. All *P* values were calculated using a two-sided Student’s *t*-test.

## Results

### Extraction of skin and microvascular features

**Fig. 4:**
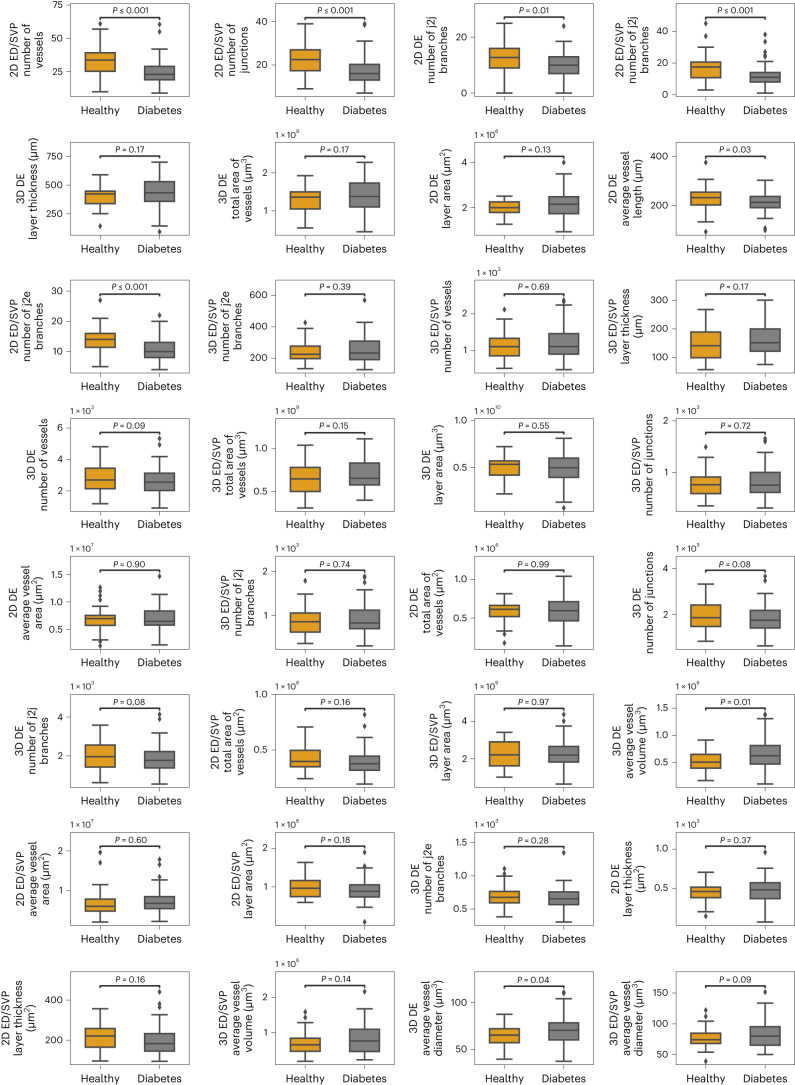
The 32 selected features of healthy volunteers and patients with diabetes. All *P* values were calculated using a two-sided Student’s *t*-test. For the boxplots, the centre line represents the median, the box limits the first and third quartiles, and the whiskers (minima and maxima) 1.5× the interquartile ranges.

Three-dimensional RSOM images from a 4 × 2 × 2 mm^3^ skin volume were obtained at 532 nm (green) from the distal anterolateral region of the dominant leg (Fig. 1a–e), ~15 cm above the ankle joint (see online Methods). Extraction of skin features from RSOM images was based on a two-step segmentation process (see online Methods and Fig. 4) using two U-NETs^26^, one for segmenting the skin layers and the other for segmenting the microvasculature. We use the term ‘microvasculature of the epidermal layer’ here to refer to the capillaries lying at the epidermal–dermal-junction level, that is, at the subpapillary vascular plexus (SVP). To generate a training dataset for the first U-NET, the acquired RSOM images were depicted as 2D maximum intensity projections (MIP), and the epidermal/SVP and dermal layers were manually segmented by two operators (A and B) with extensive experience in human RSOM images. High segmentation agreement was observed between the two operators (Table 2). High segmentation efficiency was also demonstrated for the trained U-NET when using a leave-one-subject-out (LOSO) testing (Table 2 upper panel). This first trained U-NET was then employed to automatically segment the skin layers in each individual slice of the reconstructed 3D RSOM volumes, resulting in 3D segmentations of the epidermal/SVP and dermal layers by stitching single-slice 2D segmentation into 3D volumes (see online Methods and Supplementary Fig. 1). In a second step, we processed MIP RSOM images with a tubeness filter^27^ and then applied thresholds to the segmented epidermal/SVP and dermal layers to extract maps of 2D microvasculature masks. These maps were employed to train the second U-NET exhibiting high segmentation efficiency (Table 2 lower panel). The second trained U-NET segmented the vasculature in each slice of the recorded 3D RSOM volumes. The second U-NET outperformed thresholding/filtering by leading to improved contour sharpness and reducing noise effects. The U-NET model has achieved a level of generalization beyond the specific examples it was trained on. It has also learned to extract underlying patterns and relationships from the data that enable it to make accurate predictions or decisions. Finally, using skeletonization and geometrical formulas to capture the form and orientation of the segmented microvessels, we computed a total of 64 image features.

**Table 2:** Quantification of the U-NET efficiency of the two-step segmentation process for 220 images, mean ± s.d

Step 1. Segmentation of the whole skin layer regions (epidermal/SVP layer, dermal layer)									
Segmented region	U-NET vs Operator A			U-NET vs Operator B			Operator A vs Operator B		
	Dice score	Cohen’s Kappa coefficient	Hausdorff distance	Dice score	Cohen’s Kappa coefficient	Hausdorff distance	Dice score	Cohen’s Kappa coefficient	Hausdorff distance
**Epidermal/SVP layer**	0.91 ± 0.01	0.89 ± 0.01	29.61 ± 2.18	0.87 ± 0.02	0.85 ± 0.02	34.54 ± 3.27	0.90 ± 0.02	0.89 ± 0.02	21.00 ± 2.99
**Dermal layer**	0.89 ± 0.01	0.83 ± 0.01	65.80 ± 3.29	0.86 ± 0.01	0.79 ± 0.01	73.11 ± 4.04	0.91 ± 0.01	0.87 ± 0.01	40.52 ± 3.06
**Step 2. Segmentation of the microvasculature within the skin layers (epidermal/SVP layer, dermal layer)**									
**Segmented microvasculature**	**U-NET vs Adaptive thresholding/tubeness filter**								
	**Dice score**			**Cohen’s Kappa coefficient**			**Hausdorff distance**		
**Epidermal/SVP layer**	0.90 ± 0.01			0.89 ± 0.01			23.35 ± 0.91		
**Dermal layer**	0.87 ± 0.01			0.85 ± 0.01			39.00 ± 1.86		

### Relating skin features to diabetes stage

We investigated whether different skin features are differentially affected by diabetes stage. To achieve this goal, we fed the 64 features into a random-forest (RF) classifier^28^ and identified the features with the higher descriptive power for diabetes by maximizing the ability of the feature vector to differentiate the diabetes group from the healthy group. At each tree node of the classifier, the dataset was divided into two buckets and the importance of each feature was derived according to the ‘purity’ of each bucket; the more a feature decreases the impurity, the more important it becomes^29^. The RF analysis identified the 32 features most relevant to diabetes (Figs. 2b and 4, and Table 3) by assessing their ability to accurately classify healthy individuals and patients with diabetes.

To explore the relation between diabetes stage and the 32 selected features, we categorized the patients with diabetes into three diabetes stage groups: A (diabetes without complications), B (diabetes with either neuropathy or ASCVD) and C (diabetes with neuropathy and ASCVD) (Fig. 2a). The features were also grouped into three scales of architectural detail (Figs. 2b and 4, and Table 3), that is, the microscale (<100 μm), the mesoscale (≈100–1,000 μm) and the macroscale (>1,000 μm), to study the effect of diabetes stage on each of the 32 features separately in the context of geometrical scale.

To provide a robust visual description of the 32 selected features and their changes/differences among different groups, we designed a segmental map of the features (Fig. 2c), where each ‘slot’ corresponds to a single feature and its colour intensity corresponds to the feature value, which is normalized against the maximum value on a per-feature basis. The individual vascular features were normalized only for visualization purposes. A detailed list of the 32 selected features is provided in Fig. 2d. For the effective representation of the normalized feature values, we used colour coding (Fig. 2e), where higher colour intensities (blue for microscale, orange for mesoscale and grey for macroscale) represent higher feature values within the normalized range of (0,1). Observation of each feature in relation to diabetes stage (Fig. 2e) revealed that 11 out of the 32 features demonstrate a progressive monotonic change across the three groups (A vs B, B vs C and A vs C), as elaborated in Table 1. Seven features that progressively decrease/deteriorate with diabetes stage progression belong to the highly vascularized dermal layer. In contrast, four features that increase with diabetes progression belong to the epidermal/SVP layer.

Across the patient group, the most prominent changes in features with diabetes progression occurred in the (1) decrease in the number of junction-to-junction dermal layer branches (2D), (2) the monotonic increase in the number of vessels in the epidermal/SVP layer (2D) and (3) the monotonic decrease in the number of junction-to-junction dermal layer branches (3D) (Fig. 2f). The findings in the dermal layer are consistent with the known microvascular loss due to diabetes, corroborating the findings of the analysis. The increase in vessels in the epidermal/SVP layer is of particular interest, however, and may explain how 2D methods, such as Doppler imaging, do not conclusively identify anatomical microvascular changes^30^ since they average effects from multiple skin layers and vessel directions. This detailed layer differentiation may be a critical feature of RSOM since different skin components are shown to have different responses to diabetes progression and could provide accurate tangible metrics on the effects of disease. As observed, all three features with the most prominent change with diabetes progression belong to the mesoscale (Fig. 2f).

While the influence of diabetes on several skin features is prominent in these results, none of these features appears to have significant predictive value in identifying disease stage, although their mean value decreases as diabetes progresses, as can be qualitatively observed in Fig. 2f and as quantitatively reported in Table 1 (*P* values calculated using a two-sided Student’s *t*-test). Therefore, we further examined whether the predictive value of identifying stage improves when a combination of all features is considered. To examine this premise, we developed a statistical model for ordinal classification of the RSOM images into the three diabetes stages included in the study (A, B and C), taking into account all 32 selected/diabetes-relevant features, as opposed to observing individual features. The statistical model can be expressed as:

1 $${G}^{-1}[P(Y\le j)]={a}_{j}-{\rm{X}}{\beta}$$

where **X** is the vector of the 32 features, **β** is the vector of their coefficients/weights and possible interactions, *α*_*j*_ is the threshold for each diabetes stage group/class *j* (three diabetes stage groups/classes in our case, that is, A, B and C), and *G*^−1^ is a function that links the probability that an input *P* lies below a threshold and thus belongs to a class, to the classification output (see online Methods for details). To the best of our knowledge, this analysis innovates in associating skin microvascular features to diabetes stage/progression as described by diabetes complications.

The model was fed with 75 vectors of 32 extracted features (one vector for each patient with diabetes scanned). The model classifies the 75 feature vectors and thus the patients that the vectors represent, into one of the previously described diabetes progression groups (A, B and C). The model fits both a coefficient/weight and a threshold vector to the input dataset to achieve optimal classification of each vector/patient in one of the three diabetes progression groups (Fig. 2h).

To examine the effect of different feature subgroups on the model’s performance, we examined the classification accuracy (ACC) achieved by the statistical model after removing features of the different scales (Fig. 2i). We observed that removal of different feature subgroups decreased the classification accuracy in all cases examined, indicating that all feature scales identified are significantly affected by diabetes. However, removal of the mesoscale features led to the highest decrease in classification accuracy, identifying this group of features as the most affected by diabetes progression and demonstrating the high significance of this architectural scale of detail for monitoring diabetes stage/progression. This discovery further explains the observation that the three features that exhibit the most prominent change with diabetes progression belong to the mesoscale, as do four of the seven features that degrade with diabetes progression (Table 1).

### Microangiopathy score compilation and relation to diabetes

In addition to studying the effect of diabetes on individual skin features, we also aimed to explore how a compilation of all features relates to diabetes (Fig. 3). To achieve this goal, we first classified ‘healthy’ (negative class, label equal to zero) and ‘patient with diabetes’ (positive class, label equal to one) by computing a ‘microangiopathy score’ in the range of (0,1) comprising all 32 features (Figs. 2d and 3b). An RF classifier operating on a leave-one-patient-out scheme for all 115 participants resulted in an AUC of 0.84 with a 95% confidence interval (CI) of 0.84–0.85 (Fig. 3a), an F1 score of 0.83 and a normalized Matthews correlation coefficient of 0.78 (ref. ^31^). The microangiopathy score for patients with diabetes (0.76 ± 0.13) was found to be higher than that of healthy volunteers (0.56 ± 0.16), with statistical significance of *P* < 0.001, as calculated using a two-sided Student’s *t*-test (Fig. 3c). The calculated optimal threshold for the microangiopathy score was 0.65 according to the Youden index^32^ (Methods and Fig. 3a).

Then, we computed the relative importance of each feature during the prediction/classification process, a process that allowed sorting of all features according to their classification power (Fig. 3b). For diabetes classification, that is, for the separation of healthy vs patients with diabetes, the three most important features were found to be (1) the number of vessels in the epidermal/SVP layer (2D), (2) the number of junctions in the epidermal/SVP layer (2D) and (3) the number of junction-to-junction branches in the dermal layer (2D) (Fig. 3b). The feature ‘number of junction-to-junction branches in the dermal layer (2D)’ is one of the major features describing the organization/density of the microvascular tree and appears to be strongly associated with both diabetes progression (Fig. 2) and detection (Fig. 3), being of utmost importance to the study. The junction-to-junction branches feature refers to microvessels that are connected to the same node (junction) and is indicative of rarefaction, that is, the loss of complexity of the microvascular network. We note that RSOM can uniquely identify features at the dermal layer by attaining vascular contrast, signal-to-noise ratio and penetration depths that are not offered by any other non-invasive method today.

The analysis also confirmed that mesoscale features are significantly more important than microscale features (*P* < 0.05) in separating patients with diabetes from healthy individuals. However, there was no statistical significance (*P* > 0.05, as calculated using a two-sided Student’s *t*-test) when comparing the importance of the mesoscale and macroscale features (Fig. 3e). As depicted in more detail in the comparison of the mean segmental maps (Fig. 3f) between healthy volunteers and patients with diabetes, the mesoscale features appear to be the most affected in diabetes. The latter observation is further supported by boxplots (Fig. 3g) that show the mesoscale to be the only scale significantly affected (*P* < 0.01, as calculated using a two-sided Student’s *t*-test) in patients with diabetes compared with healthy volunteers. In contrast, the values of microscale features seem to slightly increase in diabetes patients (yet statistically non-significant), while the macroscale features remain almost stable. In Fig. 4 and Table 3, we provide a detailed description of the changes observed between healthy volunteers and patients with diabetes on a per-feature basis. Lastly, we also examined the role of feature classes on the detection problem by subgrouping them to features from the epidermal and dermal layers as well as 2D and 3D features. The features in the epidermal/SVP layer exhibited an AUC of 0.69 (95% CI of 0.68–0.70), the features in the dermal layer an AUC of 0.73 (95% CI of 0.73–0.74), the 2D features an AUC of 0.67 (95% CI of 0.66–0.68) and the 3D features an AUC of 0.65 (95% CI of 0.65–0.66). Similar to the diabetes progression problem, this finding confirms that the best classification performance is achieved when all features are considered.

**Table 3:** The 32 selected features and their changes (with *P* values) between healthy volunteers and patients with diabetes

Feature number	Feature description	Feature scale	Feature change	*P* values
1	Number of vessels in the epidermal/SVP layer (2D)	Mesoscale	**↓**	<0.001***
2	Number of junctions in the epidermal/SVP layer (2D)	Mesoscale	**↓**	<0.001**
3	Number of junction-to-junction branches in the dermal layer (2D)	Mesoscale	**↓**	0.011*
4	Number of junction-to-junction branches in the epidermal/SVP layer (2D)	Mesoscale	**↓**	0.001***
5	Thickness of the dermal layer (3D)	Macroscale	**↑**	0.174
6	Total area of vessels in the dermal layer (3D)	Macroscale	**↑**	0.165
7	Area of the dermal layer (2D)	Macroscale	**↑**	0.126
8	Average vessel length in the dermal layer (2D)	Microscale	**↓**	0.031*
9	Number of junction-to-endpoint branches in the epidermal/SVP layer (2D)	Mesoscale	**↓**	<0.001***
10	Number of junction-to-endpoint branches in the epidermal/SVP layer (3D)	Mesoscale	**↑**	0.387
11	Number of vessels in the epidermal/SVP layer (3D)	Mesoscale	**↑**	0.690
12	Thickness of the epidermal/SVP layer (3D)	Macroscale	**↑**	0.170
13	Number of vessels in the dermal layer (3D)	Mesoscale	**↓**	0.093
14	Total area of vessels in the epidermal/SVP layer (3D)	Macroscale	**↑**	0.153
15	Area of the dermal layer (3D)	Macroscale	**↓**	0.546
16	Number of junctions in the epidermal/SVP layer (3D)	Mesoscale	**↑**	0.719
17	Average vessel area in the dermal layer (2D)	Microscale	**↑**	0.904
18	Number of junction-to-junction branches in the epidermal/SVP layer (3D)	Mesoscale	**↑**	0.737
19	Total area of vessels in the dermal layer (2D)	Macroscale	**↑**	0.993
20	Number of junctions in the dermal layer (3D)	Mesoscale	**↓**	0.084
21	Number of junction-to-junction branches in the dermal layer (3D)	Mesoscale	**↓**	0.084
22	Total area of vessels in the epidermal/SVP layer (2D)	Macroscale	**↓**	0.159
23	Area of the epidermal/SVP layer (3D)	Macroscale	**↑**	0.972
24	Average vessel volume in the dermal layer (3D)	Microscale	**↑**	0.010*
25	Average vessel area in the epidermal/SVP layer (2D)	Microscale	**↑**	0.595
26	Area of the epidermal/SVP layer (2D)	Macroscale	**↓**	0.181
27	Number of junction-to-endpoint branches in the dermal layer (3D)	Mesoscale	**↓**	0.279
28	Thickness of the dermal layer (2D)	Macroscale	**↑**	0.374
29	Thickness of the epidermal/SVP layer (2D)	Macroscale	**↓**	0.161
30	Average vessel volume in the epidermal/SVP layer (3D)	Microscale	**↑**	0.138
31	Average vessel diameter in the dermal layer (3D)	Microscale	**↑**	0.035*
32	Average vessel diameter in the epidermal/SVP layer (3D)	Microscale	**↑**	0.094

*P < 0.05, ***P < 0.001. All *P* values were calculated using a two-sided Student’s *t*-test.

### Control for variations with age and sex

**Fig. 5:**
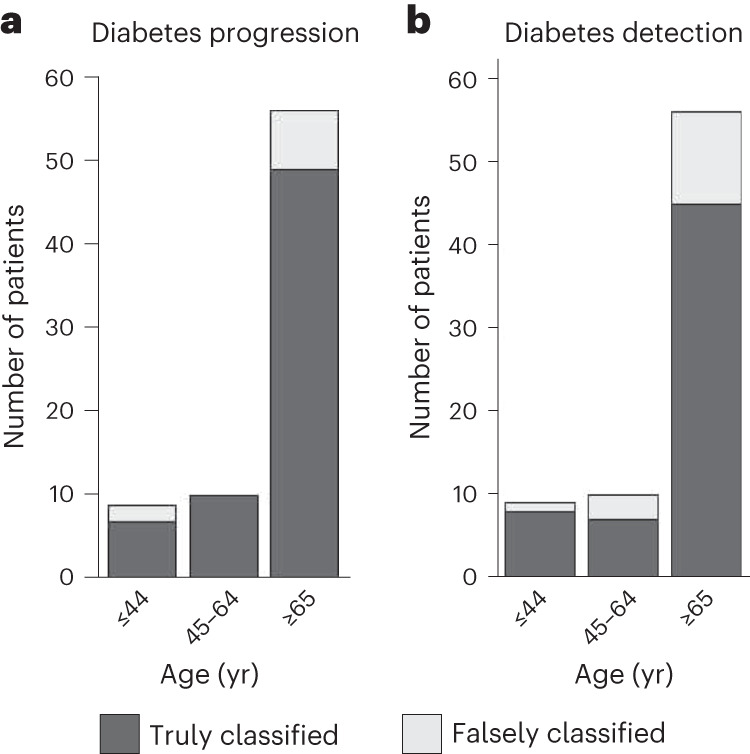
Age analysis and classification performance. **a**, For diabetes progression. **b**, For the detection of diabetes.

Since age may be a confounding factor in microvascular changes, we performed a control study to understand the effect of age in the findings. The investigation was based on three generalized linear models (GLMs)^33^, which included diabetes as a dependent binary (yes or no) variable. In particular, model 1 contained only age as an independent variable, model 2 contained both age and ‘microangiopathy score’, while model 3, apart from the age and ‘microangiopathy score’, also included their interaction term. The models were assessed by comparing their residuals (and testing their differences via a Chi-squared test), as well as the significance of each coefficient. Results showed that adding the ‘microangiopathy score’ to the model improved the explanatory capacity of the model (78.5 vs 102 predictive residuals, 0.08 vs 8.37 coefficient estimates, *P* < 0.001, as calculated using a two-sided Student’s *t*-test), while the coefficient of ‘microangiopathy score’ was statistically significant (*P* < 0.001, as calculated using a two-sided Student’s *t*-test). Furthermore, model 3 did not significantly improve model performance (75 predictive residuals, *P* = 0.063, as calculated using a two-sided Student’s *t*-test). Both studies, that is, diabetes progression and diabetes detection, were characterized by high classification rates regardless of the age group of the examined patients with diabetes (≤44, 45–64 and ≥65; Fig. 5). These results suggest that the ‘microangiopathy score’ retains its predictive value even when controlling for age, and that the interaction between the two variables does not significantly confound our results.

Furthermore, we also examined sex as a potential cofounder. In parallel with the previously described approach, model 1 contained only sex as an independent variable, model 2 contained both the sex and ‘microangiopathy score’, while model 3, apart from the sex and ‘microangiopathy score’, also included their interaction term.

The models were again assessed by comparing their residuals (and testing their differences via a Chi-squared test), as well as the significance of each coefficient. Results showed that adding the ‘microangiopathy score’ to the model improved the explanatory capacity of the model (140 vs 107 predictive residuals, 0.85 vs 8.49 coefficient estimates, *P* < 0.001, as calculated using a two-sided Student’s *t*-test), while the coefficient of ‘microangiopathy score’ was statistically significant (*P* < 0.001). Furthermore, model 3 did not further improve model performance (107 predictive residuals, *P* = 0.710, as calculated using a two-sided Student’s *t*-test).

### Nested cross-validation and external validation

Moreover, to control for overfitting and information leakage due to hyperparameter setting, that is, over-hyping effect, during the RF training, not only a nested cross-validation (CV) approach was additionally implemented, but the proposed method and the 32 selected features were also tested in an independent test set of 11 participants (online Methods). Both approaches achieved similar results to the LOSO RF classifier.

More specifically, the 30 trials of the nested CV showed that the optimal *k* for the feature selection is 31 ± 1.64 with an AUC of 0.83 ± 0.01, ACC of 0.77 ± 0.02, SNS of 0.77 ± 0.06, SPC of 0.78 ± 0.07, positive predictive value (PPV) of 0.86 ± 0.03 and negative predictive value (NPV) of 0.64 ± 0.04. Supplementary Table 9 shows all the features selected in the 30 trials sorted by their median importance. We observe that 97% of the 32 best features selected by the LOSO RF are also listed as the most important features selected by the nested CV.

Furthermore, the bootstrapped (95% bootstrapped CI) evaluation of the proposed method on an independent test set achieved an AUC of 0.87 (95% CI of 0.87–0.87), an accuracy 0.82 (95% CI of 0.82–0.82), an SNS of 0.80 (95% CI of 0.80–0.81), an SPC of 0.83 (95% CI of 0.83–0.83), a PPV of 0.80 (95% CI of 0.79–0.80) and an NPV of 0.84 (95% CI of 0.83–0.84).

## Discussion

Histological studies of the skin require invasive biopsies, which are not well suited for disseminated or longitudinal clinical studies of diabetes-related microvascular changes^8–10^. Moreover, the disruptive nature of a biopsy and the absence of blood pressure and flow in the microvessels of excised skin specimens may distort the skin architecture and affect the precision of the observations. As a consequence, the pathophysiology of diabetic microangiopathy is not routinely assessed or studied. Non-invasive imaging of the skin microvasculature in unperturbed environments opens the possibility to study skin microangiopathy in the context of different pathologies and disease stages in patients, enhancing the knowledge of the relation between diabetes and skin. RSOM did not exhibit side effects in the population imaged, took less than 1 min of scan time and was well tolerated by all patients and healthy volunteers, confirming the appropriateness of the method for clinical application. Progress with accelerating the scan time required^34^ may soon result in examination times of a few seconds, further improving applicability and throughput.

The relevance of skin measurements in diabetes healthcare has been outlined in many studies^35,36^ and was recently showcased by using photoplethysmography for the detection of diabetes^22^. Nevertheless, photoplethysmography only offers bulk measurements of skin blood volume and not a detailed study of different microvascular features. A particularly critical finding in our study is that individual high-resolution features in the skin are affected by diabetes stage in different ways. For example, there are features that decrease in density in the dermal layer while increasing in the epidermal/SVP layer as a function of stage (see section ‘Relating skin features to diabetes stage’). In this light, the RSOM approach clearly showcases the need for a detailed layer separation and highly individualized feature understanding, a task that is not achieved by photoplethysmography or laser Doppler studies.

In particular, we identified a decrease in the junction-to-junction vascular branches within the dermis to be one of the most prominent skin changes with diabetes stage (Fig. 2f). This finding is consistent with previous knowledge obtained by histological analysis of skin biopsies from diabetes patients, showing a decrease in the number of capillaries in the dermal layer and chronic capillary ischaemia^10,37,38^. These changes are known to associate with reduction in skin perfusion reserves, degraded skin biomechanics and neuropathy, leading to increased risk for disrupted skin integrity and chronic wounds^39^. Conversely, the number of branches increased in the epidermal/SVP layer. These highly detailed and 3D layer-specific insights into the unperturbed skin environment in vivo may help explain apparently contradictory observations in the literature, typically based on histological ex vivo findings, with reports ranging from microvascular density increases, thickening of the microvascular basement membrane and hyperplasia occurring with diabetes progression^9,37,40–43^, to observations of no change^9,10^ or a decrease^43,44^ in microvascular diameter.

Via our cxAI analysis, we also observed that diabetes stage affects, with higher significance, the mesoscale features, which primarily describe the organization of the skin microvascular network. Corroborating this observation, we show that the mesoscale features are the most important subgroup in the performance of the diabetes detection task via the ‘microangiopathy score’. Overall, as shown in Table 1, many individual features are affected by diabetes stage, an observation that is only made possible by the combination of cxAI and the superior image quality and high-resolution achieved by RSOM. We also observed that, although certain individual features strongly correlated with diabetes stage, the inclusion of all 32 features improved the classification accuracy, indicating that all 32 features selected are critically affected by diabetes progression. This performance may be useful in the future for redefining the ways diabetes effects are characterized in large patient populations, allowing quantitative metrics of diabetes effects and possibly leading to efficient approaches to staging and therapy monitoring.

The study was designed as an investigation of the global relevance of the relation between microangiopathy and diabetes stage, and to provide evidence on the suitability of the approach for integrating cxAI/RSOM in larger cohort studies. We acquired and analysed a patient cohort that reflects diabetes diversity including age, type-I vs type-II diabetes, different disease duration and varying macrovascular complications (Supplementary Table 1). Such a patient set revealed microvascular feature changes as a global descriptor of diabetes progression. In the future, we will explore the relevance of the RSOM features identified in this study within specific subpopulations of patients with diabetes or patient groups with targeted clinical characteristics, such as type-I vs type-II diabetes or patients with specific clinical manifestations of diabetic neuropathy to examine the predictive value of these features in assessing progression in relation to other conditions. Overall, we anticipate that the findings will stimulate the adoption of cxAI/RSOM for the study of a large range of clinical questions in diabetes.

One particular feature that may gain importance is the ability to quantify diabetes effects on skin features. This development may fulfil an unmet need in diabetes monitoring. With diabetes becoming a global epidemic^45^ affecting more than 400 million people, the ability to monitor diabetes progression in a disseminated and portable manner is critical for the efficient management of such a large patient pool and could be fundamental to prevention strategies, motivating lifestyle changes or for monitoring the efficacy of therapeutic interventions. Traditional medical tests, such as measuring blood-glucose levels after fasting^46^ or glycated haemoglobin (HbA1c) analysis, can be used for diabetes diagnosis, but these are invasive, laborious and not well suited to the monitoring of disease progression^47,48^, especially after insulin regulation interventions. In contrast to blood tests, RSOM resolves the actual effect of glucose deregulation on the state of the skin microvasculature in a frequent manner owing to its non-invasive nature and label-free operation (that is, it doesn’t require contrast agents). The medical need to assess diabetes severity and status over time is further underscored by the introduction of the adapted Diabetes Complications Severity Index (DCSI), which is designed to assess diabetes via its complications, their severity and laboratory data^14,49^. The Diabetes Severity Score (DSS) offers a metric based on parameters such as age, body mass index, duration of diabetes, presence of microvascular and macrovascular complications, the need for insulin treatment and the levels of stimulated C-peptide in blood^50^. The approach can in particular provide accurate and the most detailed readings today in terms of skin microvasculature complications, introducing the skin as a window to characterizing the effects of diabetes on the vascular system and possibly improving the accuracy of DCSI or other such indices with finer and quantitative assessment of diabetes effects on skin microvasculature compared with clinical symptoms. Therefore, the possible intended use of such technology would not be the classification of patients in groups, characterized today by crude clinical metrics, but rather the individual study of patients in a personalized manner. In other words, we foresee the use of a quantified microangiopathy score as a metric that can be integrated in healthcare to increase the precision by which the status of disease is assessed on an individual basis.

Overall, the cxAI-based RSOM framework for the assessment of skin microangiopathy could be employed as a non-invasive, label-free and highly portable method for studying and exploring diabetic skin microangiopathy in a disseminated and longitudinal fashion. The method may facilitate the sensitive detection of skin changes caused by diabetes progression and can be used to employ the skin as a window into diabetes effects and to possibly grade or monitor interventions. With the introduction of new technology, it becomes possible to formulate and address a larger number of clinical questions than what is possible today. We foresee that the integration of cxAI/RSOM with larger cohort studies could lead to additional knowledge and help establish new metrics for the management of diabetes.

## Methods

### Study design and participant preparation

In total, 115 individuals (40 healthy volunteers and 75 patients clinically diagnosed with diabetes) were included in the current study. A detailed description of the population demographics and clinical characteristics is provided in Supplementary Table 1. After thorough explanation of the aim of the study and the possible risks, each individual gave informed consent. The study was approved by the Ethics Committee of the Technical University of Munich (Protocol #109/17S). Participants were placed in a dark and quiet room with normal temperature (~23 °C) and asked to lie in a supine position. The skin of the leg was cleaned using alcohol wipes and left uncovered for at least 5 min before the planned RSOM measurement.

### RSOM setup, examination and image dataset

Individuals were scanned with the same RSOM system (see ref. ^15^ for a detailed system description) over the distal anterolateral region of the leg (~15 cm above the ankle joint) (Fig. 1a) because relevant skin lesions in diabetes may well arise at this region of the human body^39^. Furthermore, the legs are usually the position of examination for other microvascular complications of diabetes, such as diabetic peripheral neuropathy, because such complications are largely concerned with lower limbs, although the hands may also be affected in severe cases^51–53^. In brief, our customized RSOM setup was equipped with a pulsed monochromatic laser light source at 532 nm (green) for skin illumination, and a transducer with a 50 MHz central frequency for ultrasound sensing. The laser energy per pulse (~4 μJ mm^−2^) was much lower than the upper limit allowed for human use during all measurements (20 mJ cm^−2^) (ref. ^54^). For each scan, the handheld scanning probe was lightly fixed on the skin. Laser illumination was delivered via two optical fibre bundles mounted on the stable scanning probe. Two motorized stages moved the ultrasound detector to scan a rectangular skin region of 4 mm × 2 mm, acquiring thousands of single-point measurements at steps of 15 μm along the *X* axis and 7.5 μm along the *Y* axis in ~60 s (Fig. 1b,c). The depth reached was ~2 mm. For each light pulse, one ultrasound signal was recorded.

### Optoacoustic data processing

First, the recorded one-dimensional (1D) optoacoustic signals (Fig. 1c) were reconstructed into volumetric images (3D) (Fig. 1d), where the first dimension corresponded to depth (*Z* axis) and the second and third dimensions corresponded to the width (*Y* axis) and the length (*X* axis) of the scanned rectangular skin region, respectively. Before the reconstruction, a motion correction algorithm was applied on the signals^55^, which were then reconstructed independently in two frequency bands (10–40 MHz and 40–120 MHz) and finally combined into a final 3D RSOM volume^15^. The 3D volumes contained both the high-frequency (HF) and the low-frequency (LF) components of the recorded optoacoustic signals. The HF components were assigned to the red channel and the LF components to the green channel in the final RGB images, while the blue channel was left empty (Fig. 1d,e). Second, an MIP value along the *Y* axis for each frequency band was separately calculated for each volumetric image, resulting in an RGB 2D image representation containing both frequency bands (Fig. 1e). For carrying out segmentation and for visualization purposes, the images were normalized against their maximum intensity value. Normalizations were applied on the red and green channels of the 3D volume and the corresponding 2D MIP image independently.

### Deep-learning-based segmentation of skin layers and vasculature

A two-step segmentation process was used to extract features from RSOM images (Supplementary Fig. 1). First, the epidermal/SVP and dermal layers of the skin were segmented in both the reconstructed 3D and calculated 2D MIP images (Supplementary Fig. 1a,b). As already described before, considering that the epidermis does not normally contain vessels, throughout this study, the term ‘epidermal/SVP layer’ describes all structures within the approximate lower level of the epidermal layer of the skin, including small vessels (capillaries) lying directly beneath the epidermal–dermal junction (subpapillary vascular plexus), which geometrically ‘invade’ the epidermal layer from the dermal layer below. All segmentations took place in both the 2D and 3D images, allowing for the extraction of layer-specific 2D and 3D vascular features.

For the segmentation steps, two deep-learning downsized U-NETs^26^ were implemented in Keras ^56^ with a TensorFlow^57^ backend, that is, one for segmenting the skin layers (epidermal/SVP layer and dermal layer) and the other for segmenting the microvasculature within each skin layer. The two downsized U-NETs share the same architecture and each convolutional layer corresponds to 1/8 of filters in comparison with the architecture in ref. ^26^. Each U-NET had two distinct 768 × 256 × 1 outputs, ‘covering’ the segmentation masks of the epidermal/SVP and dermal layers, and the microvasculature is contained therein (microvasculature in the epidermal/SVP and dermal layers).

For the first step of the segmentation process, the reference segmentation masks for the epidermal/SVP and dermal layers were manually delineated in the 2D MIP RSOM images in consensus with two operators (A and B) with extensive experience in clinical RSOM imaging of the skin. The manually defined masks were then used to train the model of the first U-NET to automatically segment the epidermal/SVP and dermal layers for the whole dataset of 2D images (Supplementary Fig. 1c). The model output was further used to estimate the reference masks for the epidermal/SVP and dermal layers in the 3D RSOM images, since it is challenging to manually segment skin layers directly in 3D space. To this end, a mask for each skin layer was estimated for every slice along the *Y* axis by treating each slice as a 2D RSOM image. Final 3D masks were extracted by stitching the predicted single-slice 2D masks into a 3D volume. For this task, we employed the LabelMe software, an open-source annotation tool^58^.

For the second step of the segmentation process, the reference 2D vasculature masks were produced by means of adaptive thresholding^59^ of the image regions belonging to the previously predicted masks for the epidermal/SVP and dermal layers. A manual delineation of the microvascular masks was not possible due to the rich information provided by RSOM, as well as the high complexity of the vascular network in all images. Before this step, each 2D image was (1) converted to grayscale and (2) processed with a Sato tubeness filter^27^ to enhance tube-like structures, such as the blood vessels. Both the adaptive thresholding and tubeness filter were implemented in Scikit-image software^60^. The calculated 2D masks were then used to train the model of the second U-NET to automatically segment the microvasculature within the epidermal/SVP and dermal layers for all 2D images (Supplementary Fig. 1d). The vasculature masks in 3D space were again extracted from the predicted single-slice 2D masks along the *Y* axis. The reason why the second U-NET was preferred over the thresholding method is that the U-NET is designed to capture hierarchical features from the input data and learn complex patterns at different scales, enabling it to identify fine details and subtle variations in the images, which may be missed by simple thresholding or filtering approaches. In addition, unlike thresholding or filtering methods, which involve multiple steps, U-NET learns to perform segmentation in an end-to-end manner and optimize its parameters on the basis of the segmentation objective directly. U-NET utilizes a U-shaped architecture with skip connections, which helps incorporate contextual information from different layers and enables the model to understand the context of the pixels and make more informed segmentation decisions.

We followed the same deep-learning approach for each segmentation step, using a suitable set of binary masks (skin layers or microvasculature regions) for each step. Considering that the masks were binary, the segmentation tasks were considered as a pixel-wise classification problem. Thus, the pixel-wise cross-entropy loss function was used to train the two U-NETs. The pixel-wise cross-entropy loss function was defined as

2 $$L=-\mathop{\sum }\limits_{h=1}^{768}\mathop{\sum }\limits_{w=1}^{256}\left({y}_{{hw}}\mathrm{ln}\left(\,{p}_{{hw}}\right)+\left(1-{y}_{{hw}}\right)\mathrm{ln}\left(1-{p}_{{hw}}\right)\right),$$

where ${y}_{{hw}}\in \{\mathrm{0,1}\}$ is the true value of the pixel $\left(h,w\right)$ within the reference binary mask, while ${p}_{{hw}}\in \left[\mathrm{0,1}\right]$ is the prediction of the U-NET for the same pixel. During inference time, each U-NET-predicted ${p}_{{hw}}$ was further discretized to $\{\mathrm{0,1}\}$ by means of Otsu’s thresholding^61^.

The efficacy of the described AI-based two-step segmentation approach was assessed via three commonly used metrics: (1) the dice score, (2) the Hausdorff distance and (3) the Cohen’s kappa coefficient (Table 2)^62–64^. Furthermore, to check the operator independence of the efficacy of the first step of the segmentation process (manual delineation of the masks for the epidermal/SVP and dermal layers), we calculated the three evaluation metrics (Table 2) between (1) the reference masks manually defined by operator A and the segmentation result of the abovementioned U-NET model (Operator A and AI), (2) the reference masks manually defined by operator B and the segmentation result of the abovementioned U-NET model (Operator B and AI) and (3) the manually defined reference masks of the two operators (Operator A and Operator B). According to the calculated metrics, all comparisons yielded similarly good results (see Results). The combination of Operator A and AI showed slightly better performance (Table 2) and was therefore selected for the rest of the analysis, including the second step of the segmentation process (microvasculature in the epidermal/SVP and dermal layers).

The final 2D and 3D masks of the vasculature in the epidermal/SVP and dermal layers were further skeletonized (Supplementary Fig. 1e) and the produced vascular networks’ skeletons were utilized to extract vascular features for each 2D MIP and 3D image (Supplementary Fig. 1f). To overcome the scarcity of the available training data, a LOSO training scheme was employed, where each patient (or healthy volunteer) was left out and the remaining data were split into training (90%) and validation (10%) sets and finally used to train a U-NET model instance. For the deep-learning segmentation of skin layers and vasculature, we used 220 images of 129 participants. However, 21 images of 14 participants were excluded from the diabetes detection and progression problems because of multiple comorbidities of these patients. Thus, 115 distinct U-NET model instances were trained and each one was used to predict the segmentation mask of its corresponding test case. Each U-NET instance was trained for a maximum number of 200 epochs using the Adam optimization method^65^, saving the model parameters for each epoch, followed by improvement on the validation loss. In addition, early stopping was employed, so that the training process of each U-NET model instance was prematurely stopped if no improvement in the validation loss was achieved for 40 consecutive epochs.

### Extraction of skin and microvascular features

As a next step, we extracted clinically relevant and explainable features that describe the morphology of the skin layers and the microvasculature to understand the effects of microangiopathy. These features were extracted from the eight previously segmented masks; that is, from the 2D and 3D masks of the whole epidermal/SVP and dermal layers, and the 2D and 3D masks corresponding to the microvasculature of the epidermal/SVP and dermal layers. In total, 64 features were extracted for each RSOM image, corresponding to (1) the epidermal/SVP region of the 2D MIP images, (2) the dermal region of the 2D MIP images, (3) the epidermal/SVP region of the 3D images and (4) the dermal region of the 3D images. Thus, each of these subgroups was represented by 16 features described in detail in Supplementary Table 2. The calculation of each vascular feature was based on the calculation of the skeleton of the microvasculature for each 2D MIP and 3D image. Specifically, each region (epidermal/SVP and dermal) was processed using the skeletonize function of the Scikit-image software^60,66^ and all the features were automatically calculated using the skeleton’s functions of the Skan library^67^ implemented in Python. For the calculation of the features, raw data of RSOM 2D and 3D images were used, and no normalization was applied.

This feature set was used to both optimize the description of the skin microvasculature and ultimately maximize the information content and explainability of the calculated microangiopathy score (Supplementary Fig. 1f) for each participant, which is equal to ‘the probability that the measurement corresponds to a patient’.

### Calculation of the microangiopathy score and diabetes detection

The 64 vascular features were fed into an RF classifier (Supplementary Fig. 1f)^28^, which generated a ‘microangiopathy score’ in the range of (0,1). If more than one image was available for a participant, the mean value of the calculated single-image microangiopathy scores was taken as the microangiopathy score of the participant. The final microangiopathy scores of all participants were employed to classify them either as ‘healthy’ (negative class, label equal to 0) and ‘patient with diabetes’ (positive class, label equal to 1). The higher values of the microangiopathy score corresponded to diabetes (>0.65) and the lower values to healthy (≤0.65) (see Results). Similar to the segmentation deep-learning model applied above, a LOSO CV was employed for all 115 participants to achieve an unbiased estimation of the calculated score for each participant. In every iteration of this validation scheme, each of the 115 patients (or volunteers) was left out of the training dataset as a test case and the rest were used to train the RF classifier. Different classifiers and algorithms were tested (RF classifier, decision tree classifier, multilayer perceptron classifier and logistic regression) and the classification performance is reported using the AUC. However, the RF classifier outperformed the abovementioned classifiers, hence it was selected for the diabetes classification problem. The Scikit-learn^68^ implementation of the RF was employed, using a total of 100 trees in the forest and no maximum depth of the tree.

Furthermore, to ensure that no information leakage or data overfitting occurred due to hyperparameter setting, that is, to ensure no over-hyping effect, during the training for the diabetes classification problem, we implemented a nested CV method^69,70^. The nested CV used different train/validation/test set splits, and we ran the nested CV procedure 30 times. In each trial, a LOSO scheme was implemented, where the ‘GridSearchCV’ implemented in Scikit-learn^68^ optimized the number of estimators and the maximum number of features for the RF classifier. The optimization was based on an independent inner set, which was split into 4 consecutive folds (KFold cross-validator in Scikit-learn^68^). In addition, in each iteration we used the ‘SelectKBest’ algorithm to find how many features achieved the best classification.

An advantage of the used RF classifier is its embedded feature selection capability, which assigns a score to each feature on the basis of how the feature reduces the overall Gini impurity^29^. In this manner, features that perform better in terms of classification are assigned higher scores by the RF. This capability allowed the definition of a subset ($k$ best features) of the initially extracted 64 features, which provided the best classification performance between the ‘healthy’ and ‘diabetes’ groups. Furthermore, a ‘SelectKBest’ feature selection algorithm in Scikit-learn^68^ was employed before the RF to reduce the initial feature-vector dimensionality and select the $k$ best features. We benchmarked our approach for all $k$ within the interval (10, 64), while considering various feature groups, including those exclusive to the epidermal/SVP layer, dermal layer, 2D features and 3D features.

The output of the whole process, that is, the microangiopathy score, was assigned from the trained classifier by computing the predicted class probability of the under-test image. After the LOSO CV, the microangiopathy score was computed by averaging the probabilities per participant; thus, it ranges from 0 to 1, and is higher than the calculated optimal classification threshold (0.65) for patients with diabetes and lower for healthy volunteers. The standard deviation of the microangiopathy score was calculated using the standard deviation of the score across the individual trees in the RF. The selection of the optimal $k$ ($k=32$) and the performance of different feature groups were evaluated using the AUC values (Supplementary Fig. 2)^71^, summarizing the method’s predictive performance across all discretization thresholds of the positive class probability. The optimal decision/classification threshold (0.65) was calculated by means of the Youden index, a well-established metric for rating diagnostic tests^32^. Nevertheless, the ACC, SNS, SPC, PPV and NPV of the classifier were calculated as: ACC = (TP + TN)/(TP + TN + FP + FN), SNS = TP/(TP + FN), SPC = TN/(TN + FP), PPV = TP/(TP + FP), NPV = TN/(TN + FN), where TP, TN, FP and FN correspond to the number of true positive, true negative, false positive and false negative predictions, respectively.

To explore the presence of possible bias in the cohort of 40 healthy volunteers vs 75 patients with diabetes, we assessed the outcome of the microangiopathy score-based classification for combinations of 40 patients belonging to the initial group of the 75 patients with diabetes. Since the number of all possible 40-sized patient subgroups were 3 × 10^21^, we performed the corresponding calculations between the group of the 40 healthy volunteers and a random set of 5,000 different 40-sized patient subgroups. A clear difference (*P* < 0.001, as calculated using a two-sided Student’s *t*-test) was identified between the calculated microangiopathy scores for the ‘healthy’ group (median 0.45 with a 95% CI of 0.40–0.50) and the ‘patient’ group (median 0.63 with a 95% CI of 0.58–0.67). This negates any possible effect from the dataset imbalance, increasing the credibility of the produced classification performance.

Moreover, to further validate our method, we also used an independent dataset for testing. This dataset was collected from 5 patients with diabetes and 6 healthy volunteers, from a study approved by the Ethics Committee of the Technical University of Munich (Protocol #326/19S). We trained an RF classifier on the initial cohort of 115 participants (75 patients with diabetes and 40 healthy volunteers) using the 32 selected best features as an input, and we evaluated the model on the independent test set. Furthermore, the 95% CIs for the results were calculated by using a bootstrapping approach^72,73^ due to the small test set.

### Clinically relevant subgroup analysis

To further evaluate the efficacy of the previous classification task, we conducted a subgroup analysis within the diabetes group by defining 6 subgroups on the basis of parameters with clinical relevance (Supplementary Fig. 4f–k): (1) The HbA1c level. To convert this into a binary variable, the value of 6.5% was selected as a cut-off point, as it represents the diagnostic level of diabetes (>6.5%)^74^. Moreover, in patients with diagnosed diabetes, HbA1c reflects the level of glycaemic control or the efficacy of an applied therapy during the last 3 months. In our cohort, we included 75 patients with diabetes: 58 with HbA1c ≥ 6.5% and 17 with HbA1c < 6.5%. (2) The total neuropathy score, which is the sum of the neuropathy symptom score (NSS) and neuropathy disability score (NDS)^75^. NSS and NDS are two clinical scores used to assess peripheral diabetic neuropathy, a very common complication of diabetes, which is also thought to be of microvascular origin^4^. A total neuropathy score (NSS + NDS) of 8 or more is thought to be an indicator of diabetic neuropathy^76^. In the current study, we were able to record the total neuropathy score for 51 patients, of which 37 patients were characterized by a total neuropathy score ≥8 and 14 patients by a score <8. (3) The presence of macrovascular ASCVD, that is, of peripheral arterial disease (PAD) and coronary artery disease (CAD), which are both possible complications of diabetes^4^. From the 75 patients included, 52 had no PAD or CAD, 7 had only PAD, 12 had only CAD and 4 had both. (4) The participant’s age. We categorized the patients into three age groups of 18–44, 45–64 and >65 yr, which are common age groups in the analysis of epidemiological trends of diabetes in the general population^74^. From the 75 patients included in the current study, 9 belonged to the group of 18–44 yr, 10 to the group of 45–64 yr and 56 to the group of >65 yr. (5) The type of diabetes. Our cohort included 25 patients with type-I diabetes and 50 patients with type-II diabetes. (6) The duration of diabetes. The 75 patients were categorized into 4 groups with different durations of diabetes: 23 of them had diabetes for 0–10 yr at the examination timepoint, 20 for 11–20 yr, 12 for 21–30 yr and 20 for >30 yr.

After splitting the diabetes group into subgroups on the basis of the aforementioned splitting scheme, the proportion of truly and falsely classified individuals per subgroup was calculated and plotted in the form of stacked bar plots (Supplementary Fig. 4f–k).

### Categorization of the selected features into different scales of detail

The multiscale character of our approach was realized by categorizing the selected microvascular features into three different scales of detail: the ‘microscale’, the ‘mesoscale’ and the ‘macroscale’. The microscale group included features describing the characteristics of single microvessels, such as the vessel diameter or length. Thus, the microscale features provide a zoomed-in, single-vessel representation of the skin microvasculature. The mesoscale group refers to features describing small, localized networks or neighbourhoods of microvessels. The mesoscale group included features that provide information about the organization or ‘complexity’ of the imaged microvascular network, such as the number of vessels and junctions. Finally, the macroscale group was assigned vascular features describing rough characteristics of the images of the skin region, such as the total vessel area or the thickness of the epidermal/SVP and dermal layer. A detailed description of the features belonging to each scale of detail is provided in Fig. 2d and Supplementary Table 2.

### Diabetes progression analysis

To model the progression of diabetes, we categorized the 75 patients with diabetes into 3 groups (Fig. 2a): A for patients with diabetes without neuropathy or ASCVD (*n*_A_ = 22), B for patients with diabetes and only neuropathy or only ASCVD but not both (*n*_B_ = 29) and C for patients with diabetes and both neuropathy and ASCVD (*n*_C_ = 24). Assuming that the 3 defined groups describe ordinal data (from A to C with increasing disease severity), we applied a cumulative link model for ordinal regression analysis/classification^77^ on the basis of the 32 selected features that yielded the best classification/diabetes detection performance via the microangiopathy score (see Results). The actual model parameters were automatically selected from a pool containing the 32 features, as well as their interactions. This was achieved using a two-process selection scheme. In the first process, a standard stepwise model selection was performed using the build-in step function of R, with both directions (stepwise removal or addition) available at each step. This process used the Akaike Information Criterion (AIC) and is therefore based on parsimony. In the second process, further elimination of variables was performed on the basis of a leave-one-out evaluation of each model’s accuracy. Thus, the variable selection approach aimed at a balance between parsimony and predictive performance.

For consistency with the diabetes detection part of the analysis, we estimated the ‘importance’ of each selected feature and each scale of extracted features (microscale, mesoscale, macroscale) in the outcome of the diabetes progression classification model. In this section, we defined importance as the decrease in the classification accuracy caused after the removal from the model of each group/scale of features. Results are presented in the form of boxplots of the median and 85% of all combinations of the features belonging to each scale (see Results).

### Reporting summary

Further information on research design is available in the Nature Portfolio Reporting Summary linked to this article.

### Supplementary information

Supplementary Information Supplementary methods, figures and tables.

Reporting Summary

Supplementary Data 1 Calculated feature values per scan.

Supplementary Data 2 Importances of the features for the diabetes detection problem.

Supplementary Data 3 Feature values for the independent test dataset.

Supplementary Data 4 Microangiopathy score per patient for the diabetes detection problem.

## Data Availability

The post-processed data (calculated features) for each individual are provided as Supplementary Information. The trained models and the optoacoustic data are available for research purposes from the corresponding author on reasonable request.

## Code availability

The code for the data analysis is available on Zenodo at 10.5281/zenodo.10047634.
