# Supplementary material for: Dermal features derived from optoacoustic tomograms via machine learning correlate microangiopathy phenotypes with diabetes stage
Source: Nat Biomed Eng. 2023 Dec 4;7(12):1667–82. doi: 10.1038/s41551-023-01151-w (PMC10727986; doi:10.1038/s41551-023-01151-w)
Supplement: Supplementary file 1 — Supplementary methods, figures and tables. [file 41551_2023_1151_MOESM1_ESM.pdf]

nature biomedical engineering

Article https://doi.org/10.1038/s41551-023-01151-w

Dermal features derived from optoacoustic tomograms via machine learning correlate microangiopathy phenotypes with diabetes stage

In the format provided by the authors and unedited

Supplementary methods

Selection the optimal features number

For the diabetes detection problem, in order to select the k best features out of the 64 initially extracted, which achieve the highest performance, a 'SelectKBest' feature selection algorithm was employed before the RF classifier. For all k within the interval [10, 64], the k best features were fed into the RF and their performance was evaluated using the AUC value. As shown in Supplementary Fig. 2, when $k = 32$ features were selected, the classifier performed the highest AUC of 0.84 with a 95% CI [0.84, 0.85].

Clustering of the 32 selected features

We also explored if our approach preferentially selected features belonging to one dermal layer or spatial dimension. For this reason, we performed for the 32 selected features a k-means clustering for three clusters, corresponding to the three different scales of detail (micro-, meso-, macroscale). As shown in the clustering tree (Supplementary Fig. 3), 14 of the features are 2D (extracted from the calculated MIP image), 18 are 3D (extracted from the initially reconstructed volumetric image), 15 correspond to the dermal layer and 17 to the epidermal/SVP layer. These results show that no 'imbalances' in type of layer or in spatial dimension were present in the characteristics of the 32 selected features.

Classification efficacy in clinically relevant subgroups

To further evaluate the performance of the proposed classification methods for diabetes progression and detection, we conducted two further subgroup analyses for the diabetes group. The different subgroups were selected using parameters with clinical relevance.

For the diabetes progression classification, as observed in Supplementary Fig. 4a, the model truly classifies 52 (90% with a 95% CI [89.4%, 90.1%]) of the 58 patients with HbA1c > 6.5% and 14 (82% with a 95% CI [82.0%, 82.9%]) of the 17 patients with HbA1c < 6.5%. Supplementary Fig. 4b demonstrates that our approach truly classifies 33 (89% with a 95% CI [88.5%, 89.2%]) of the 37 patients with NDS+NSS ≥ 8 and 13 (93% with a 95% CI [92.5%, 93.1%]) of the 14 patients with NDS + NSS < 8. Moreover, Supplementary Fig. 4c shows that we are able to truly classify 46 (88% with a 95% CI [88.2%, 89.0%]) of the 52 patients without PAD or CAD, 6 (86% with a 95% CI [85.6%, 86.4%]) of the 7 patients only with PAD, 10 (83% with a 95% CI [82.8%, 83.7%]) of the 12 patients with CAD solely and 4 (100%) of the 4 patients with both PAD and CAD. Supplementary Fig. 4d provides the results of the subgroup analysis based on the type of diabetes. As shown, we are able to truly classify 22 (88% with a 95% CI [87.4%, 88.2%]) of the 25 type-I-diabetes patients and 44 (88% with a 95% CI [87.3%, 88.1%]) of the 50 patients with type-II-diabetes. Finally, our method efficiently classifies 17 (74% with a 95% CI [73.0%, 74.2%]) out of 23 patients with diabetes duration of 0-10 years, 18 (90% with a 95% CI [89.9%, 90.6%]) out of 20 patients with diabetes duration of 11-20 years, 12 (100%) out of 12 patients with diabetes of 21-30 years and 19 (95% with a 95% CI [94.8%, 95.3%]) out of 20 patients with diabetes duration of more than 30 years (Supplementary Fig. 4e).

As observed in Supplementary Fig. 4f, the suggested approach detects 45 (77% with a 95% CI [76.8%, 77.9%]) of the 58 patients with HbA1c > 6.5% and 15 (88% with a 95% CI [87.8%, 88.6%]) of the 17 patients with HbA1c < 6.5%. Supplementary Fig. 4g shows that our algorithm detects 30 (81% with a 95% CI [80.5%, 81.5%]) of the 37 patients with NDS+NSS ≥ 8 and 12 (86% with a 95% CI [85.5%, 86.3%]) of the 14 patients with NDS + NSS < 8. Furthermore, Supplementary Fig. 4h demonstrates that we are able to detect 42 (81% with a 95% CI [80.2%, 81.2%]) of the 52 patients without PAD or CAD, 5 (71% with a 95% CI [71.0%, 72.2%]) of the 7 patients with PAD, 10 (83% with a 95% CI [82.7%, 83.7%]) of the 12 patients with CAD and 3 (75% with a 95% CI [74.7%, 75.9%]) of the 4 patients with both PAD and CAD. Supplementary Fig. 4i summarizes the outcomes of the subgroup analysis based on the type of diabetes. Based on the abovementioned cxAI-based approach, we are able to detect 21 (84% with a 95% CI [83.3%, 84.2%]) of the 25 type-I-diabetes patients and 39 (78% with a 95% CI [77.7%, 78.7%]) of the 50 patients with type-II-diabetes. Finally, the newly developed microangiopathy score efficiently detects 19 (83% with a 95% CI [81.9%, 82.9%]) out of 23 patients with diabetes duration of 0-10 years, 14 (70% with a 95% CI [69.3%, 70.4%]) out of 20 patients with diabetes duration of 11-20 years, 8 (67% with a 95% CI [66.2%, 67.3%]) out of 12 patients with diabetes of 21-30 years and 19 (95% with a 95% CI [94.7%, 95.2%]) out of 20 patients with diabetes duration of more than 30 years (Supplementary Fig. 4k). The outcomes show that the efficacy of both classification methods for the corresponding tasks-at-hand (diabetes progression and detection) is not only high but also unaffected by several parameters with clinical relevance.

Clinically relevant subgroup analysis of the segmental feature maps

To explore the changes of the newly developed segmental maps with reference to the different clinical diabetes-related parameters, we performed a relevant subgroup analysis for the diabetes group. Our results are summarized in Supplementary Fig. 5 (a-g).

With reference to age (Supplementary Fig. 5b), we observe that the mesoscale features are significantly decreased (p<0.05, as calculated by a two-sided Student's t-test) in patients with diabetes more than 64 years old, compared to younger patients (Age ≤ 44 years). For example, the greatest statistically significant decrease (p<0.05, as calculated by a two-sided Student's t-test) occurs in the number of junction-to-endpoint branches in the 3D representation of the dermis (feature #27, mesoscale) in the patients older than 65 years, compared to patients with age in the range of 45-64 years. A detailed list of the per-feature p-values among the different age subgroups of the patients is provided in Supplementary Table 3.

Regarding HbA1c subgroups (Supplementary Fig. 5c) in patients with diabetes (HbA1c < 6.5% vs. HbA1c ≥ 6.5%), our analysis shows an increase in the values of the extracted vascular features at all levels of detail in patients with HbA1c ≥ 6.5%, Nevertheless, the observed increase is not statistically significant. In contrast, the mesoscale feature 'number of junction-to-junction branches in the 2D representation of the dermis' is characterized by a significant decrease (p<0.05, as calculated by a two-sided Student's t-test) in patients with HbA1c ≥ 6.5%, compared to patients with HbA1c < 6.5%. A detailed list of the per-feature p-values between the two HbA1c subgroups is provided in Supplementary Table 4.

With reference to the duration of diabetes (years from first diagnosis of the disease) we observe a clear, yet statistically not-significant, degradation of all feature scales (micro-, meso- and macroscale) with increasing duration of diabetes (Supplementary Fig. 5d, Supplementary Table 5). Indicatively, for the microscale the average vessel length in the 2D visualization of the dermis (feature #8) is significantly (p<0.01, as calculated by a two-sided Student's t-test) decreased in patients who have had diabetes for more than 30 years, compared to patients with 11-20 years since the first diagnosis. In the mesoscale, the number of junction-to-junction branches in the 2D image of the dermis (feature #3) is characterized by a significant (p<0.05, as calculated by a two-sided Student's t-test) decrease in patients with more than 30 years with diabetes, compared to patients who were diagnosed with diabetes within the last decade (0-10 years diabetes duration). Finally, in the macroscale, the total area of vessels in the 3D representation of the epidermal/SVP layer (feature #14) seems to be significantly decreased in patients suffering from diabetes for more than 30 years, compared to the ones having diabetes in the last 21-30 years. A list of the per-feature p-values among all diabetes duration subgroups is provided in Supplementary Table 5.

As observed in Supplementary Fig. 5e and Supplementary Table 6, the feature with the most significant difference (p<0.05, as calculated by a two-sided Student's t-test) increased in the patients with increased (neuropathy score ≥ 8) between the two neuropathy groups for the patients with diabetes (total neuropathy score < or ≥ 8) belongs to the microscale (average vessel volume in the 3D representation of the dermis, feature #24). In general, though, the micro- and mesoscale features demonstrate a clear, yet statistically non-significant decrease with increasing neuropathy scores (≥ 8 compared to < 8) while the macroscale remains relatively stable between the two neuropathy groups.

Supplementary Fig. 5f and Supplementary Table 7 report the changes in the extracted vascular features in the micro-, meso- and macroscale among the subgroups of patients with diabetes based on the presence and extent of atherosclerotic cardiovascular disease. In the microscale we observe that the patients with PAD have significantly (p < 0.05, as calculated by a two-sided Student's t-test) lower microvascular feature values compare to the patients with CAD. In the mesoscale, we observe a statistically significant (p<0.05, as calculated by a two-sided Student's t-test) increase of the relevant features in patients with both PAD and CAD, compared to the patients without ASCVD. The latter observation is also extended to the macroscale features (p<0.01, as calculated by a two-sided Student's t-test).

Last but not least, Supplementary Table 8 provides a detailed description of the segmental maps for the type-of-diabetes subgroups, as presented in Supplementary Fig. 5g. On the one hand, the microscale features of the patients with type II diabetes show a discreet but non-significant decrease compared to the patients with type I diabetes. On the other hand, both the mesoscale and macroscale RSOM-extracted features are clearly lower (p<0.05 for the mesoscale, as calculated by a two-sided Student's t-test) in the patients with type I diabetes than the ones with type II diabetes.

Reproducibility of the measurements

To evaluate the reproducibility of the measurements, two operators measured five healthy volunteers on both right and left legs (Supplementary Fig. 6a) leading to 20 new RSOM measurements. The operators employed fiduciary skin markers (black ink markings on the skin) to ensure that the same position is measured. The study was approved by the Ethics Committee of the Technical University of Munich (Protocol #323/21 S). Applying the cxAI developed herein, we extracted again the 32 most important features from the RSOM images acquired. Since the differences found between measurements by operator 1 and 2 were not normally distributed (skewness=-1.122; Supplementary Fig. 6b) we applied first a logarithmic transformation to the difference data (skewness=-0.187, Supplementary Fig. 6c) (ref. [1]) and then computed the Bland-Altman-Diagram (Supplementary Fig. 6d). We found that 96.25% of the measurements lie within $\pm 1.96SD$ (standard deviation) of the mean difference, showcasing excellent reproducibility, especially when considering the large field of view (4 mm x 2 mm) examined and the high resolution achieved (10-30 μm). Paired t-tests were further employed to test for the significance of differences between operators. These tests were performed for each of the 32 variables independently. In the resulting datasets and feature vectors, no differences were found to be statistically significant ($p > 0.05$, as calculated by a two-sided Student's t-test).

Our corresponding analysis demonstrated good reproducibility for the current study. Further studies are needed in order to extensively explore the reproducibility of the RSOM technology across many different systems with other imaging specifications and hardware configurations.

Correlation analysis of extracted skin features

To investigate the correlation coefficients among the 32 selected features, we calculated the corresponding correlation matrix (Supplementary Fig. 7). As observed, some pairs of calculated features are characterized by a correlation equal to one. For example, the number of vessels and the number of junctions are highly correlated, as expected. Such features might seem to be 'redundant' but remain, nevertheless, important descriptors of the microanatomy/geometry of the microvascular network.

Meaningfulness of vascular features

To explore the meaningfulness of the extracted vascular features, two experts with long experience in RSOM imaging manually counted the dermal-layer vessels and vessel junctions in five 2D MIP images. The human annotations performed a high correlation with the features calculated by our algorithm. More specifically, the Pearson correlation for the calculation of the number of vessels is: Algorithm output vs. Operator #1 = 0.999, Algorithm output vs. Operator #2 = 0.989, while for the number of junctions is: Algorithm output vs. Operator #1 = 0.9948, Algorithm output vs. Operator #2 = 0.981.

The reason why the experts conducted this analysis only for these two features is that these ones can manually be calculated, while other features, such as the vessel's diameter or length, are impossible to be calculated in such manner, especially since the vasculature visualized by RSOM within the skin is very rich. Moreover, we conducted this analysis only in the 2D space, because the manual count of the corresponding features in the 3D space is practically impossible, due to the extremely large number (>1000) of them within each RSOM image.

References

1. Giavarina, D., *Understanding Bland Altman Analysis.* Biochemia medica, 2015. **25**(2): p. 141-151.

Supplementary figures

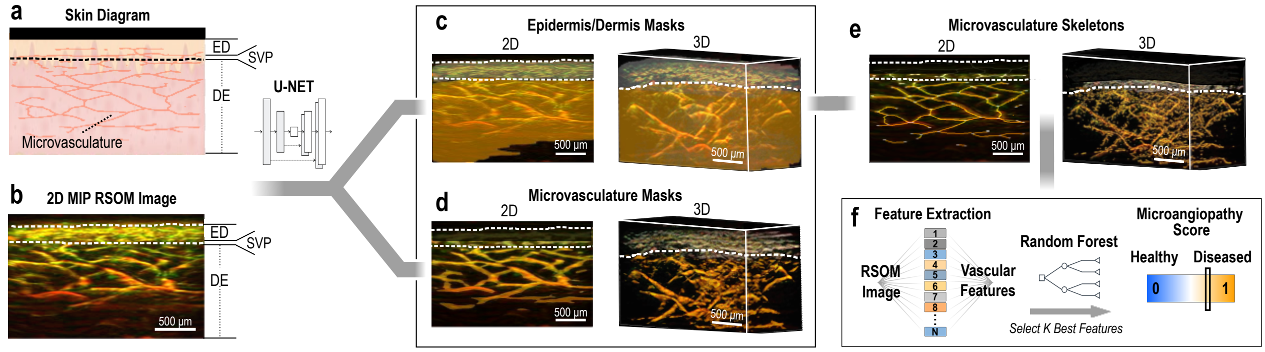

Supplementary Fig. 1 / Machine learning-based segmentation of RSOM images and calculation of microangiopathy score. (a) Schematic representation of the RSOM image in **(b)** showing the microanatomy of the imaged skin region. **(b)** Initial 2D RSOM image used as input to the U-NET. The white dashed lines delineate the epidermal/SVP layer. **(c)** Representative result of the U-NET skin layer segmentation (epidermal/SVP and dermal layers) in the 2D and 3D space. In the 3D image, only the borderline between the epidermal/SVP and dermal layers is delineated for visualization purposes. **(d)** Corresponding result of the skin microvasculature segmentation (epidermal/SVP and dermal layers) in the 2D and 3D space. **(e)** Final vasculature skeleton of the skin in 2D and 3D space employed for the extraction of explainable vascular features and the calculation of the microangiopathy score. **(f)** RSOM-extracted vascular features are fed into a RF classifier, which finally assigns a microangiopathy score to each RSOM image. Scale bars: 500 µm.

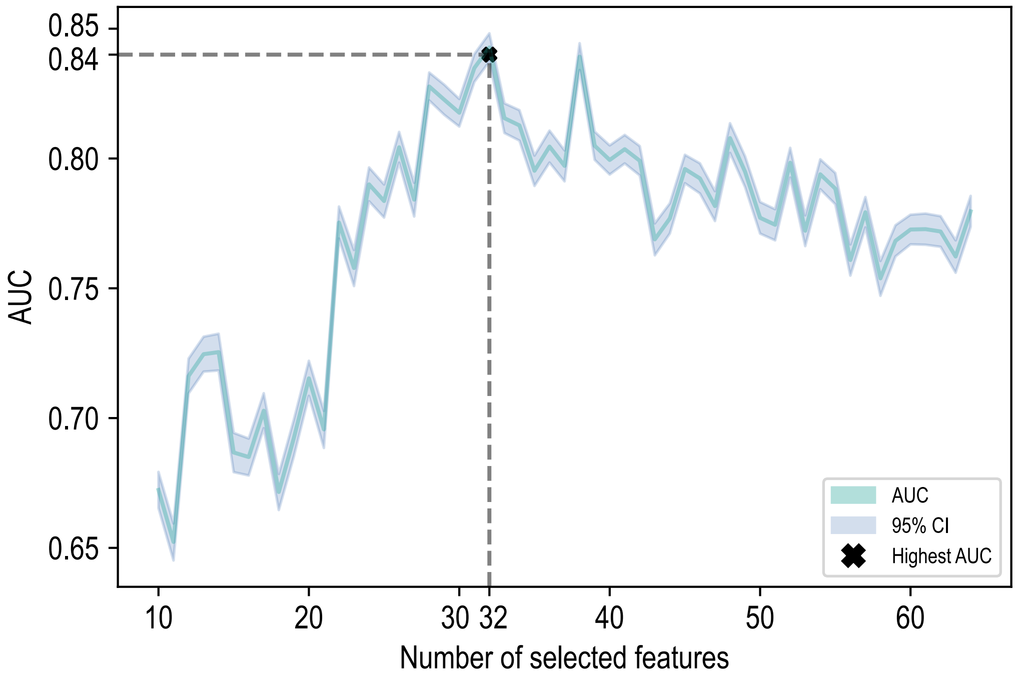

Supplementary Fig. 2 | Evaluation of the RF classifier for all number of features within the interval [10, 64] for the diabetes detection problem. The highest AUC of 0.84 with a 95% CI [0.84, 0.85] is achieved when 32 features are selected.

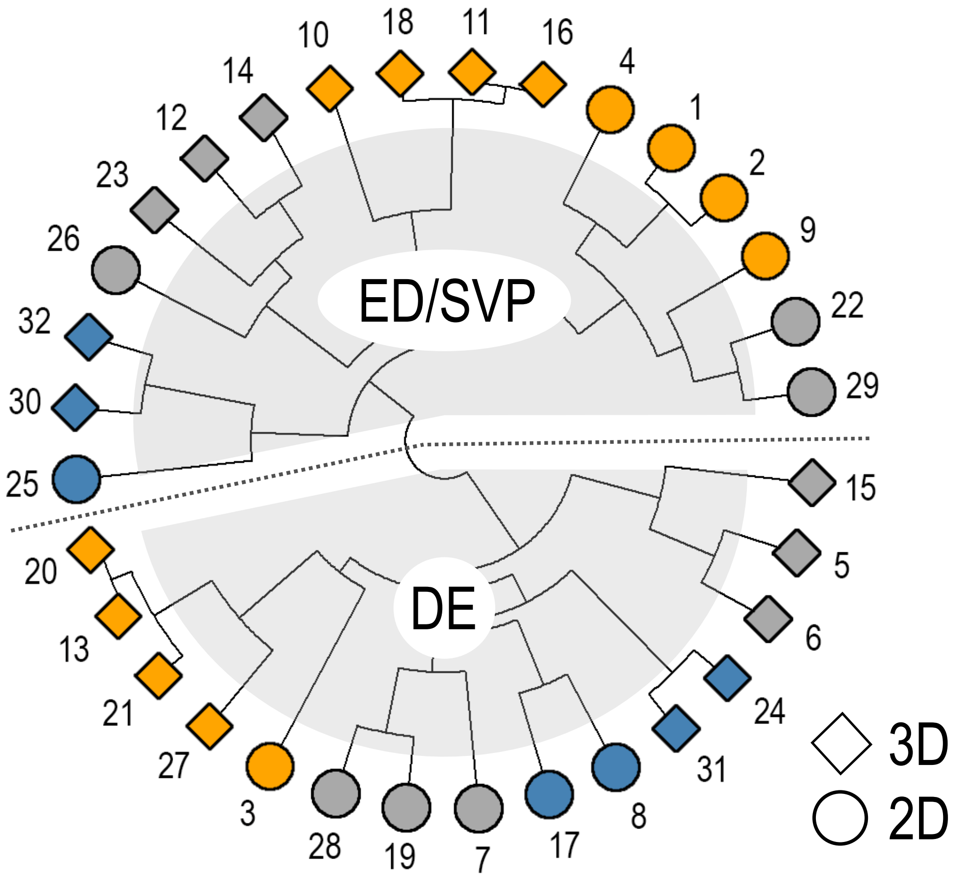

Supplementary Fig. 3 | Clustering tree based on K-means clustering of the 32 selected 2D and 3D RSOM-extracted skin features. ED/SVP, epidermal layer / subpapillary vascular plexus; DE, dermal layer.

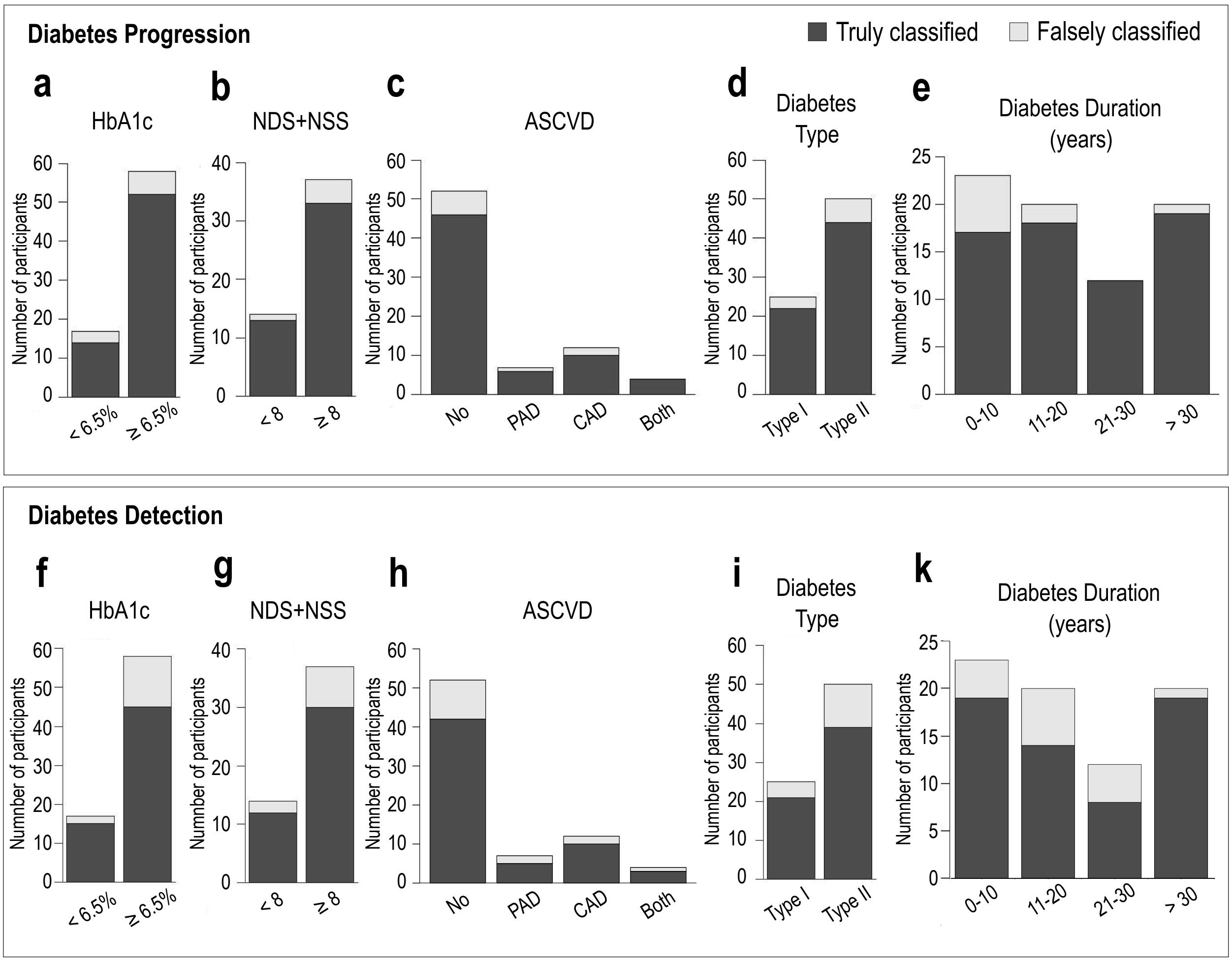

Supplementary Fig. 4 | Subgroup analysis for the diabetes progression (a-e) and detection (f-k) classification with reference to: (a, f) The HbA1c level, **(b, g)** the total neuropathy score level, **(c, h)** the presence and type of macrovascular ASCVD, **(d, i)** the type of diabetes and **(e, k)** the duration of diabetes

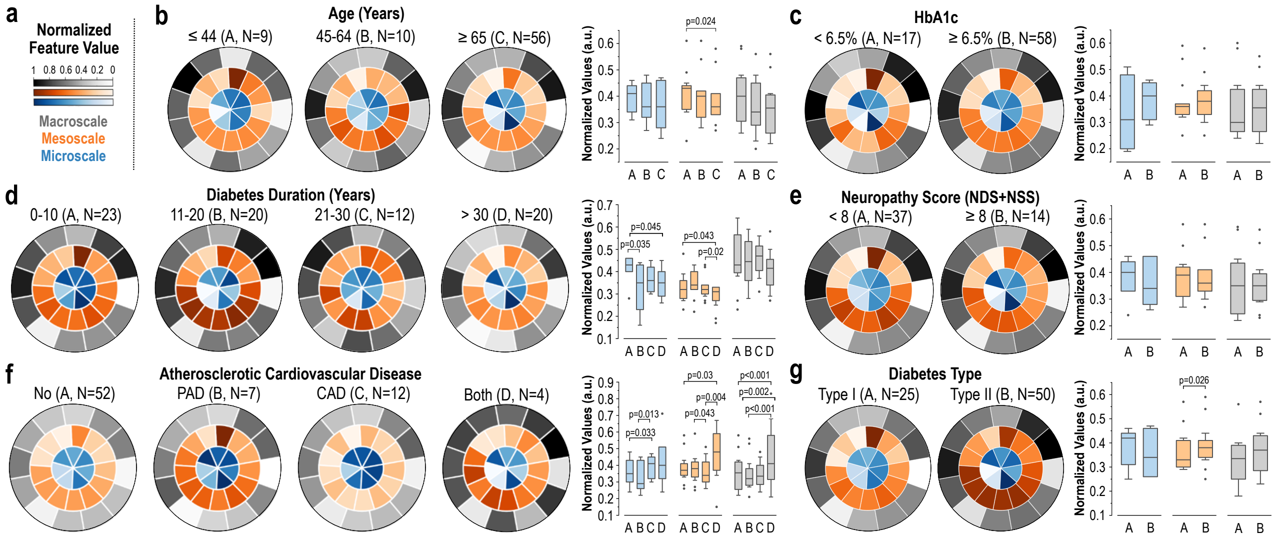

Supplementary Fig. 5 I Segmental feature maps and statistical analysis of the normalized values, against the maximum value on a per-feature basis of the different subgroups. (a) Descriptive legend. **(b)** Age subgroups. **(c)** HbA1c subgroups. **(d)** Diabetes duration subgroups. **(e)** Total neuropathy score subgroups. **(f)** Atherosclerotic cardiovascular disease subgroups. **(g)** Diabetes type subgroups. p>0.05: not shown. All p-values are shown in Supplementary Tables 3-8 and are calculated by a two-sided Student's t-test. For the boxplots, the centre line represents the median, the box limits the first and third quartiles, and the whiskers (minima and maxima) the 1.5 × interquartile ranges. The intensity of each segment corresponds to the normalized mean value of the feature for each group, as described by the three colourbars (blue, orange, grey).

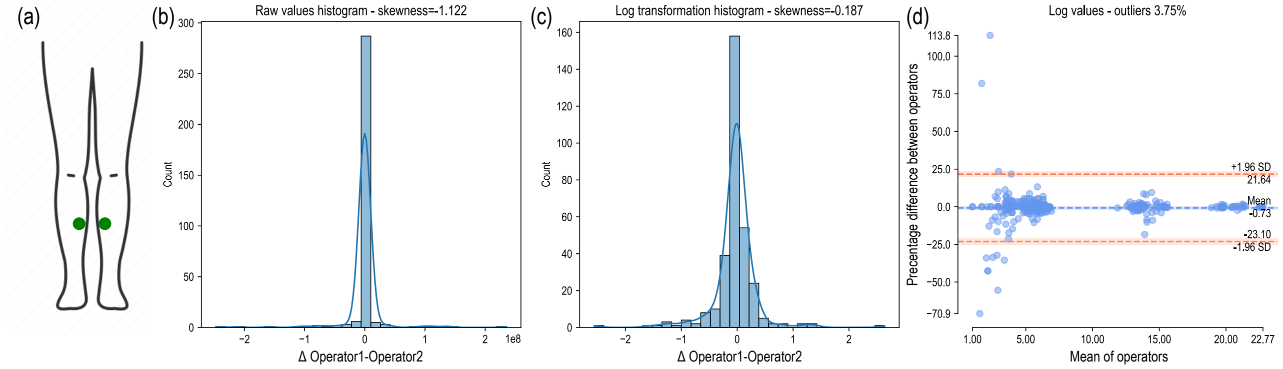

Supplementary Fig. 6 | Reproducibility of the measurements (a) Measured positions for reproducibility measurements by two operators, **(b)** Distribution plot of differences between measurements by operator 1 and 2, **(c)** Distribution plot of differences between logarithmic transformed measurements by operator 1 and 2, **(d)** Bland and Altman plot for logarithmic transformed data. SD, standard deviation.

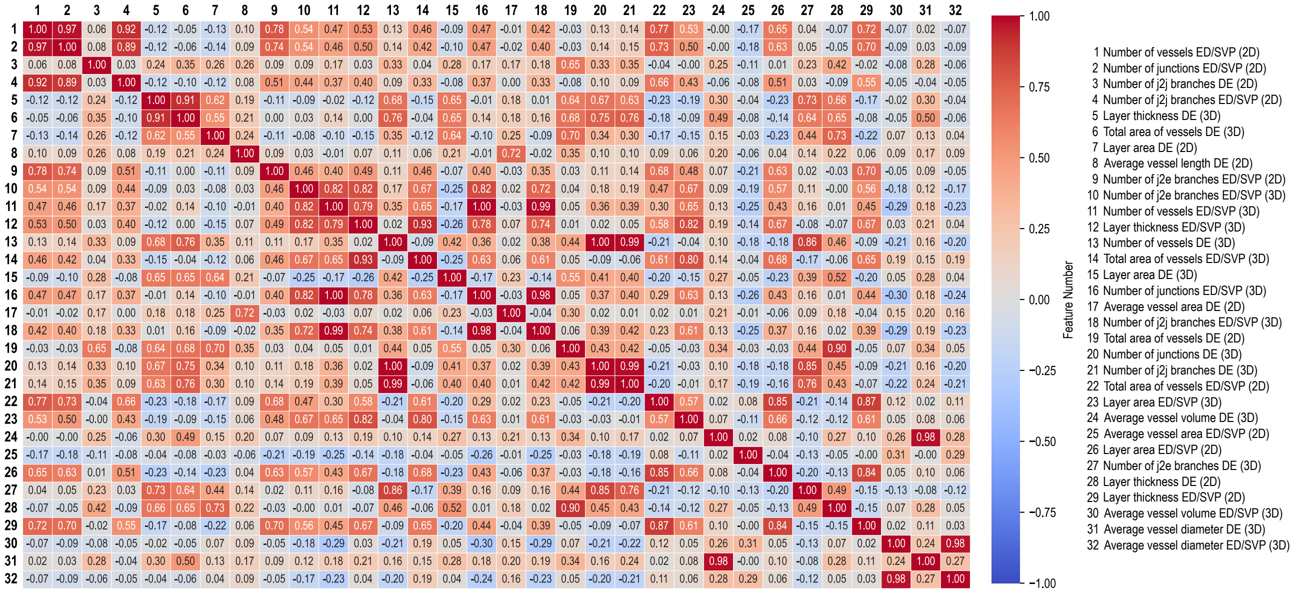

Supplementary Fig. 7 | Correlation matrix showing the correlation coefficients among the 32 selected features. ED/SVP, epidermal layer / subpapillary vascular plexus; DE, dermal layer; j2j, junction-to-junction; j2e, junction-to-endpoint.'

Supplementary tables

Supplementary Table 1. | Population demographics and clinical characteristics. s.d., standard deviation, M: male, F: Female, NS: Neuropathy Score.

<table>
<tr><th></th><th>Mean age ± s.d. (years)</th><th>Sex (M/F)</th><th>Type of diabetes</th><th colspan="2">Neuropathy</th><th colspan="2">ASCVD</th><th>HbA1C</th><th>Medical therapy</th></tr>
<tr><td rowspan="4">Patients with Diabetes</td><td rowspan="4">68.52 ± 14.95</td><td rowspan="4">49/26</td><td rowspan="3">Type I: 25</td><td rowspan="3">Yes: 51</td><td>NS ≥ 8: 37</td><td rowspan="3">Yes: 26</td><td>PAD: 7</td><td rowspan="3">≥ 6.5%: 58</td><td>Insulin: 36</td></tr>
<tr><td rowspan="2">NS < 8: 14</td><td>CAD: 12</td><td rowspan="2">Oral Therapy: 11</td></tr>
<tr><td>Both: 4</td></tr>
<tr><td>Type II: 50</td><td colspan="2">No: 24</td><td colspan="2">No: 49</td><td>< 6.5%: 17</td><td>Both: 73</td></tr>
<tr><td>Healthy Volunteers</td><td>43.90 ± 16.11</td><td>15/25</td><td></td><td colspan="2"></td><td colspan="2"></td><td></td><td></td></tr>
</table>

Supplementary Table 2 I The 16 RSOM-features for each 'region/dimension' subgroup.

Feature name	Feature description
**Vasculature intensity**	Sum of all pixel intensities in the grayscale RSOM image for the pixels belonging to the microvasculature mask
**Layer intensity**	Sum of all pixel intensities for the pixels belonging to the corresponding skin layer mask
**Total area of vessels**	Number of pixels belonging to the microvasculature mask
**Layer area**	Number of pixels belonging to the skin layer mask
**Number of vessels**	Number of all vessels/branches of the microvasculature skeleton
**Number of junctions**	Number of all vessels/branches of the microvasculature skeleton
**Junctions-to-vessels ratio**	Ratio of the total number of junctions to the total number of microvessels
**Number of junction-to-endpoint branches**	Total number of junction-to-endpoint branches in the microvasculature skeleton
**Number of junction-to-junction branches**	Total number of junction-to-junction branches in the microvasculature skeleton
**Layer thickness**	Average size (in μm) of all columns along the skin layer mask
**Vascular density**	Total number of vessels per skin layer area
**Junction density**	Total number of junctions per skin layer area
**Average vessel length**	Average length of all vessels within a skin layer
**Vessel diameter**	Average diameter of all vessels within a skin layer
**Vascular length-to-width ratio**	Average ratio of the vascular length to the vascular diameter within a layer
**Average vessel area/volume**	Average area for all vessels within a skin layer for 2D images and the average volume for all vessels within a skin layer for 3D images

Supplementary Table 3 l *p*-values for each selected feature among the age subgroups of patients with diabetes, *$p < 0.05$. All p-values are calculated by a two-sided Student's t-test.

Feature number	Feature description	Feature scale	Age ≤ 44 (N=9) vs. age 45–64 (N=10)	Age ≤ 44 (N=9) vs. age > 65 (N=56)	Age 45–64 (N=10) vs. age > 65 (N=56)
1	Number of vessels in the epidermal/SVP layer (2D)	Mesoscale	0.391	0.128	0.227
2	Number of junctions in the epidermal/SVP layer (2D)	Mesoscale	0.298	0.125	0.359
3	Number of junction-to-junction branches in the dermal layer (2D)	Mesoscale	***0.036**	***0.047**	0.242
4	Number of junction-to-junction branches in the epidermal/SVP layer (2D)	Mesoscale	0.335	0.174	0.405
5	Thickness of the dermal layer (3D)	Macroscale	0.263	0.204	0.441
6	Total area of vessels in the dermal layer (3D)	Macroscale	0.482	0.431	0.455
7	Area of the dermal layer (2D)	Macroscale	0.248	0.185	0.417
8	Average vessel length in the dermal layer (2D)	Microscale	0.447	0.266	0.308
9	Number of junction-to-endpoint branches in the epidermal/SVP layer (2D)	Mesoscale	0.472	0.129	0.108
10	Number of junction-to-endpoint branches in the epidermal/SVP layer (3D)	Mesoscale	0.460	0.487	0.417
11	Number of vessels in the epidermal/SVP layer (3D)	Mesoscale	0.410	0.482	0.393
12	Thickness of the epidermal/SVP layer (3D)	Macroscale	0.220	0.174	0.403
13	Number of vessels in the dermal layer (3D)	Mesoscale	0.297	0.472	0.161
14	Total area of vessels in the epidermal/SVP layer (3D)	Macroscale	0.090	***0.039**	0.369
15	Area of the dermal layer (3D)	Macroscale	0.272	0.259	0.372
16	Number of junctions in the epidermal/SVP layer (3D)	Mesoscale	0.419	0.500	0.387
17	Average vessel area in the dermal layer (2D)	Microscale	0.450	0.186	0.209
18	Number of junction-to-junction branches in the epidermal/SVP layer (3D)	Mesoscale	0.406	0.483	0.397
19	Total area of vessels in the dermal layer (2D)	Macroscale	0.446	0.445	0.483
20	Number of junctions in the dermal layer (3D)	Mesoscale	0.305	0.475	0.175
21	Number of junction-to-junction branches in the dermal layer (3D)	Mesoscale	0.419	0.384	0.260
22	Total area of vessels in the epidermal/SVP layer (2D)	Macroscale	0.324	0.202	***0.045**
23	Area of the epidermal/SVP layer (3D)	Macroscale	0.335	0.152	0.372
24	Average vessel volume in the dermal layer (3D)	Microscale	0.294	0.226	0.073
25	Average vessel area in the epidermal/SVP layer (2D)	Microscale	0.184	0.382	0.095
26	Area of the epidermal/SVP layer (2D)	Macroscale	0.225	0.421	0.099
27	Number of junction-to-endpoint branches in the dermal layer (3D)	Mesoscale	0.082	0.263	***0.024**
28	Thickness of the dermal layer (2D)	Macroscale	0.488	0.349	0.360
29	Thickness of the epidermal/SVP layer (2D)	Macroscale	0.293	0.317	0.084
30	Average vessel volume in the epidermal/SVP layer (3D)	Microscale	0.291	0.305	0.423
31	Vessel diameter in the dermal layer (3D)	Microscale	0.277	0.292	0.079

32	Vessel diameter in the epidermal/SVP layer (3D)	Microscale	0.350	0.299	0.498
All microscale features			0.404	0.471	0.431
All mesoscale features			0.067	***0.024**	0.334
All macroscale features			0.330	0.219	0.354

Supplementary Table 4 I *p*-values for each selected feature among the HbA1c subgroups of patients with diabetes, *$p < 0.05$. All p-values are calculated by a two-sided Student's t-test.

**Feature number**	**Feature description**	**Feature scale**	**HbA1c <6.5% (N=17) vs. HbA1c ≥ 6.5% (N=58)**
1	Number of vessels in the epidermal/SVP layer (2D)	Mesoscale	0.426
2	Number of junctions in the epidermal/SVP layer (2D)	Mesoscale	0.389
3	Number of junction-to-junction branches in the dermal layer (2D)	Mesoscale	***0.036**
4	Number of junction-to-junction branches in the epidermal/SVP layer(2D)	Mesoscale	0.455
5	Thickness of the dermal layer (3D)	Macroscale	0.262
6	Total area of vessels in the dermal layer (3D)	Macroscale	0.201
7	Area of the dermal layer (2D)	Macroscale	0.430
8	Average vessel length in the dermal area (2D)	Microscale	0.259
9	Number of junction-to-endpoint branches in the epidermal/SVP layer (2D)	Mesoscale	0.336
10	Number of junction-to-endpoint branches in the epidermal/SVP layer (3D)	Mesoscale	0.235
11	Number of vessels in the epidermal/SVP layer (3D)	Mesoscale	0.126
12	Thickness of the epidermal/SVP layer (3D)	Macroscale	0.055
13	Number of vessels in the dermal layer (3D)	Mesoscale	0.397
14	Total area of vessels in the epidermal/SVP layer (3D)	Macroscale	0.060
15	Area of the dermal layer (3D)	Macroscale	0.181
16	Number of junctions in the epidermal/SVP layer (3D)	Mesoscale	0.136
17	Average vessel area in the dermal layer (2D)	Microscale	0.349
18	Number of junction-to-junction branches in the epidermal/SVP layer (3D)	Mesoscale	0.118
19	Total area of vessels in the dermal layer (2D)	Macroscale	0.187
20	Number of junctions in the dermal layer (3D)	Mesoscale	0.408
21	Number of junction-to-junction branches in the dermal layer (3D)	Mesoscale	0.335
22	Total area of vessels in the epidermal/SVP layer (2D)	Macroscale	0.247
23	Area of the epidermal/SVP layer (3D)	Macroscale	0.146
24	Average vessel volume in the dermal layer (3D)	Microscale	0.226
25	Average vessel area in the epidermal/SVP layer (2D)	Microscale	0.317
26	Area of the epidermal/SVP layer (2D)	Macroscale	0.221
27	Number of junction-to-endpoint branches in the dermal layer (3D)	Mesoscale	0.461
28	Thickness of the dermal layer (2D)	Macroscale	0.203
29	Thickness of the epidermal/SVP layer (2D)	Macroscale	0.103
30	Average vessel volume in the epidermal/SVP layer (3D)	Microscale	0.139
31	Vessel diameter in the dermal layer (3D)	Microscale	0.223
32	Vessel diameter in the epidermal/SVP layer (3D)	Microscale	0.124
All microscale features			0.413
All mesoscale features			0.431
All macroscale features			0.294

Supplementary Table 5 l *p*-values for each feature among the subgroups with refer to the duration of diabetes, *$p < 0.05$, **$p < 0.01$. All p-values are calculated by a two-sided Student's t-test.

Feature number	Feature description	Feature scale	0-10 (N=23) vs. 11-20 (N=20)	0-10 (N=23) vs. 21-30 (N=12)	0-10 (N=23) vs. > 30 (N=20)	11-20 (N=20) vs. 21-30 (N=12)	11-20 (N=20) vs. > 30 (N=20)	21-30 (N=12) vs. > 30 (N=20)
1	Number of vessels in the epidermal/SVP layer (2D)	Mesoscale	0.489	0.348	0.185	0.362	0.183	0.065
2	Number of junctions in the epidermal/SVP layer (2D)	Mesoscale	0.334	0.277	0.350	0.429	0.191	0.109
3	Number of junction-to-junction branches in the dermal layer (2D)	Mesoscale	0.178	0.127	***0.013**	0.385	0.119	0.208
4	Number of junction-to-junction branches in the epidermal/SVP layer (2D)	Mesoscale	0.364	0.373	0.051	0.261	0.095	***0.017**
5	Thickness of the dermal layer (3D)	Macroscale	0.289	0.329	0.357	0.179	0.173	0.439
6	Total area of vessels in the dermal layer (3D)	Macroscale	0.327	0.187	0.199	0.111	0.103	0.410
7	Area of the dermal layer (2D)	Macroscale	0.440	0.476	0.376	0.439	0.348	0.433
8	Average vessel length in the dermal layer (2D)	Microscale	0.267	0.245	***0.026**	0.126	****0.009**	0.180
9	Number of junction-to-endpoint branches in the epidermal/SVP layer (2D)	Mesoscale	0.499	0.376	0.471	0.399	0.477	0.399
10	Number of junction-to-endpoint branches in the epidermal/SVP layer (3D)	Mesoscale	0.330	0.461	0.070	0.340	0.063	0.133
11	Number of vessels in the epidermal/SVP layer (3D)	Mesoscale	0.200	0.247	0.213	0.425	0.090	0.093
12	Thickness of the epidermal/SVP layer (3D)	Macroscale	0.479	0.166	0.052	0.222	0.085	****0.009**
13	Number of vessels in the dermal layer (3D)	Mesoscale	0.155	0.148	0.172	0.064	***0.049**	0.402
14	Total area of vessels in the epidermal/SVP layer (3D)	Macroscale	0.330	0.271	***0.014**	0.181	0.054	****0.006**
15	Area of the dermal layer (3D)	Macroscale	0.446	0.260	0.342	0.324	0.412	0.360
16	Number of junctions in the epidermal/SVP layer (3D)	Mesoscale	0.191	0.251	0.238	0.409	0.095	0.107
17	Average vessel area in the dermal layer (2D)	Microscale	0.157	0.486	***0.026**	0.272	0.143	0.109

18	Number of junction-to-junction branches in the epidermal/SVP layer (3D)	Mesoscale	0.188	0.208	0.263	0.446	0.105	0.100
19	Total area of vessels in the dermal layer (2D)	Macroscale	0.413	0.192	0.078	0.202	0.093	0.381
20	Number of junctions in the dermal layer (3D)	Mesoscale	0.155	0.143	0.168	0.062	***0.048**	0.401
21	Number of junction-to-junction branches in the dermal layer (3D)	Mesoscale	0.162	0.183	0.134	0.084	***0.042**	0.494
22	Total area of vessels in the epidermal/SVP layer (2D)	Macroscale	0.434	0.174	0.281	0.231	0.238	0.073
23	Area of the epidermal/SVP layer (3D)	Macroscale	0.298	0.125	***0.022**	0.263	***0.014**	****0.009**
24	Average vessel volume in the dermal layer (3D)	Microscale	0.133	0.197	0.486	0.448	0.152	0.216
25	Average vessel area in the epidermal/SVP layer (2D)	Microscale	0.059	0.262	0.145	***0.018**	0.254	*0.04324
26	Area of the epidermal/SVP layer (2D)	Macroscale	0.295	0.233	0.458	0.367	0.343	0.270
27	Number of junction-to-endpoint branches in the dermal layer (3D)	Mesoscale	0.187	0.078	0.344	***0.036**	0.123	0.155
28	Thickness of the dermal layer (2D)	Macroscale	0.233	0.254	0.289	0.154	0.146	0.445
29	Thickness of the epidermal/SVP layer (2D)	Macroscale	0.344	0.094	0.328	0.201	0.494	0.165
30	Average vessel volume in the epidermal/SVP layer (3D)	Microscale	0.058	0.061	0.149	0.463	0.254	0.264
31	Vessel diameter in the dermal layer (3D)	Microscale	0.163	0.258	0.481	0.419	0.145	0.236
32	Vessel diameter in the epidermal/SVP layer (3D)	Microscale	0.053	0.106	0.247	0.365	0.157	0.251
All microscale features			***0.035**	0.108	***0.045**	0.141	0.269	0.261
All mesoscale features			0.365	0.414	***0.043**	0.439	0.022	***0.020**
All macroscale features			0.446	0.431	0.160	0.356	0.171	0.063

Supplementary Table 6 | *p*-values for each feature between the two neuropathy subgroups, *$p < 0.05$. All *p*-values are calculated by a two-sided Student's t-test.

**Feature number**	**Feature description**	**Feature scale**	**NDS + NSS < 8 (N=14) vs. NDS + NSS ≥ 8 (N=37)**
1	Number of vessels in the epidermal/SVP layer (2D)	Mesoscale	0.351
2	Number of junctions in the epidermal/SVP layer (2D)	Mesoscale	0.289
3	Number of junction-to-junction branches in the dermal layer (2D)	Mesoscale	0.061
4	Number of junction-to-junction branches in the epidermal/SVP layer (2D)	Mesoscale	0.486
5	Thickness of the dermal layer (3D)	Macroscale	0.278
6	Total area of vessels in the dermal layer (3D)	Macroscale	0.473
7	Area of the dermal (layer 2D)	Macroscale	0.490
8	Average vessel length in the dermal layer (2D)	Microscale	0.398
9	Number of junction-to-endpoint branches in the epidermal/SVP layer (2D)	Mesoscale	0.227
10	Number of junction-to-endpoint branches in the epidermal/SVP layer (3D)	Mesoscale	0.388
11	Number of vessels in the epidermal/SVP layer (3D)	Mesoscale	0.421
12	Thickness of the epidermal/SVP layer (3D)	Macroscale	0.352
13	Number of vessels in the dermal layer (3D)	Mesoscale	0.181
14	Total area of vessels in the epidermal/SVP layer (3D)	Macroscale	0.468
15	Area of the dermal layer (3D)	Macroscale	0.359
16	Number of junctions in the epidermal/SVP layer (3D)	Mesoscale	0.465
17	Average vessel area in the dermal layer (2D)	Microscale	0.332
18	Number of junction-to-junction branches in the epidermal/SVP layer (3D)	Mesoscale	0.440
19	Total area of vessels in the dermal layer (2D)	Macroscale	0.443
20	Number of junctions in the dermal layer (3D)	Mesoscale	0.185
21	Number of junction-to-junction branches in the dermal layer (3D)	Mesoscale	0.176
22	Total area of vessels in the epidermal/SVP layer (2D)	Macroscale	0.472
23	Area of the epidermal/SVP layer (3D)	Macroscale	0.392
24	Average vessel volume in the dermal layer (3D)	Microscale	***0.023**
25	Average vessel area in the epidermal/SVP layer (2D)	Microscale	0.134
26	Area of the epidermal/SVP layer (2D)	Macroscale	0.284
27	Number of junction-to-endpoint branches in the dermal layer (3D)	Mesoscale	0.230
28	Thickness of the dermal layer (2D)	Macroscale	0.490
29	Thickness of the epidermal/SVP layer (2D)	Macroscale	0.248
30	Average vessel volume in the epidermal/SVP layer (3D)	Microscale	0.427
31	Vessel diameter in the dermal layer (3D)	Microscale	***0.040**
32	Vessel diameter in the epidermal/SVP layer (3D)	Microscale	0.406
All microscale features			0.263
All mesoscale features			0.405
All macroscale features			0.452

Supplementary Table 7 | *p*-values for each feature among all atherosclerotic cardiovascular disease subgroups, *$p < 0.05$, **$p < 0.01$, ***$p < 0.001$. All *p*-values are calculated by a two-sided Student's t-test.

Feature number	Feature description	Feature scale	No (N=52) vs. PAD (N=7)	No (N=52) vs. CAD (N=12)	No (N=52) vs. Both (N=4)	PAD (N=7) vs. CAD (N=12)	PAD (N=7) vs. Both (N=4)	CAD (N=12) vs. Both (N=4)
1	Number of vessels in the epidermal/SVP layer (2D)	Mesoscale	0.431	0.409	0.360	0.498	0.399	0.384
2	Number of junctions in the epidermal/SVP layer (2D)	Mesoscale	0.459	0.453	0.215	0.385	0.195	0.207
3	Number of junction-to-junction branches in the dermal layer (2D)	Mesoscale	0.372	0.185	0.241	0.382	0.386	0.426
4	Number of junction-to-junction branches in the epidermal/SVP layer (2D)	Mesoscale	0.378	0.490	0.352	0.280	0.436	0.316
5	Thickness of the dermal layer (3D)	Macroscale	0.168	0.439	***0.022**	0.223	0.146	0.070
6	Total area of vessels in the dermal layer (3D)	Macroscale	0.280	0.327	0.083	0.237	0.258	0.096
7	Area of the dermal layer (2D)	Macroscale	0.287	0.345	***0.024**	0.423	0.121	0.062
8	Average vessel length in the dermal layer (2D)	Microscale	0.193	0.197	0.362	0.172	0.413	0.267
9	Number of junction-to-endpoint branches in the epidermal/SVP layer (2D)	Mesoscale	0.415	0.178	0.413	0.275	0.317	0.179
10	Number of junction-to-endpoint branches in the epidermal/SVP layer (3D)	Mesoscale	0.394	0.279	0.060	0.228	0.131	****0.005**
11	Number of vessels in the epidermal/SVP layer (3D)	Mesoscale	0.489	0.114	0.133	0.179	0.199	****0.006**
12	Thickness of the epidermal/SVP layer (3D)	Macroscale	0.442	0.423	***0.048**	0.498	0.114	***0.015**
13	Number of vessels in the dermal layer (3D)	Mesoscale	0.353	0.127	0.348	0.083	0.457	0.108
14	Total area of vessels in the epidermal/SVP layer (3D)	Macroscale	0.404	0.437	0.064	0.456	0.128	***0.031**
15	Area of the dermal layer (3D)	Macroscale	0.353	0.362	0.258	0.465	0.252	0.229
16	Number of junctions in the epidermal/SVP layer (3D)	Mesoscale	0.485	0.111	0.149	0.171	0.214	0.008
17	Average vessel area in the dermal layer (2D)	Microscale	0.430	0.113	0.486	0.207	0.477	0.287
18	Number of junction-to-junction branches in the epidermal/SVP layer (3D)	Mesoscale	0.485	0.098	0.177	0.185	0.234	***0.010**
19	Total area of vessels in the dermal layer (2D)	Macroscale	0.320	0.477	0.077	0.371	0.164	0.118
20	Number of junctions in the dermal layer (3D)	Mesoscale	0.339	0.125	0.359	0.077	0.482	0.115

21	Number of junction-to-junction branches in the dermal layer (3D)	Mesoscale	0.380	0.102	0.374	0.083	0.468	0.092
22	Total area of vessels in the epidermal/SVP layer (2D)	Macroscale	0.350	0.270	0.383	0.062	0.248	0.473
23	Area of the epidermal/SVP layer (3D)	Macroscale	0.341	0.097	***0.035**	0.307	0.050	***0.010**
24	Average vessel volume in the dermal layer (3D)	Microscale	0.367	0.198	0.256	0.393	0.386	0.414
25	Average vessel area the epidermal/SVP layer (2D)	Microscale	0.426	0.475	0.193	0.427	0.258	0.143
26	Area of the epidermal/SVP layer (2D)	Macroscale	0.478	0.293	0.481	0.360	0.499	0.376
27	Number of junction-to-endpoint branches in the dermal layer (3D)	Mesoscale	0.382	0.283	0.295	0.254	0.387	0.199
28	Thickness of the dermal layer (2D)	Macroscale	0.263	0.424	****0.004**	0.276	0.052	***0.026**
29	Thickness of the epidermal/SVP layer (2D)	Macroscale	0.439	0.373	0.280	0.276	0.213	0.272
30	Average vessel volume in the epidermal/SVP layer (3D)	Microscale	0.369	***0.021**	0.307	0.090	0.430	0.096
31	Vessel diameter in the dermal layer (3D)	Microscale	0.376	0.255	0.318	0.438	0.436	0.450
32	Vessel diameter in the epidermal/SVP layer (3D)	Microscale	0.345	***0.022**	0.287	0.077	0.423	0.079
All microscale features			0.268	***0.033**	0.417	***0.013**	0.419	0.085
All mesoscale features			0.295	0.126	***0.030**	***0.043**	0.068	*****0.004**
All macroscale features			0.239	0.225	****0.002**	0.437	*****<0.001**	*****<0.001**

Supplementary Table 8 | *p*-values for each feature between the two subgroups with refer to diabetes type, $*p < 0.05$. All *p*-values are calculated by a two-sided Student's t-test.

Feature number	Feature description	Feature scale	Type I (N=25) vs. Type II (N=50)
1	Number of vessels in the epidermal/SVP layer (2D)	Mesoscale	0.123
2	Number of junctions in the epidermal/SVP layer (2D)	Mesoscale	0.244
3	Number of junction-to-junction branches in the dermal layer (2D)	Mesoscale	0.308
4	Number of junction-to-junction branches in the epidermal/SVP layer (2D)	Mesoscale	0.125
5	Thickness of the dermal layer (3D)	Macroscale	***0.047**
6	Total area of vessels in the dermal layer (3D)	Macroscale	0.061
7	Area of the dermal layer (2D)	Macroscale	0.248
8	Average vessel length in the dermal layer (2D)	Microscale	0.176
9	Number of junction-to-endpoint branches in the epidermal/SVP layer (2D)	Mesoscale	0.197
10	Number of junction-to-endpoint branches in the epidermal/SVP layer (3D)	Mesoscale	***0.040**
11	Number of vessels int eh epidermal/SVP layer (3D)	Mesoscale	0.074
12	Thickness of the epidermal/SVP layer (3D)	Macroscale	0.112
13	Number of vessels in the dermal layer (3D)	Mesoscale	0.131
14	Total area of vessels in the epidermal/SVP layer (3D)	Macroscale	0.281
15	Area of the dermal layer (3D)	Macroscale	0.270
16	Number of junctions in the epidermal/SVP layer (3D)	Mesoscale	0.082
17	Average vessel area in the dermal layer (2D)	Microscale	0.245
18	Number of junction-to-junction branches in the epidermal/SVP layer (3D)	Mesoscale	0.098
19	Total area of vessels in the dermal layer (2D)	Macroscale	0.370
20	Number of junctions in the dermal layer (3D)	Mesoscale	0.133
21	Number of junction-to-junction branches in the dermal layer (3D)	Mesoscale	0.147
22	Total area of vessels in the epidermal/SVP layer (2D)	Macroscale	0.217
23	Area of the epidermal/SVP layer (3D)	Macroscale	0.138
24	Average vessel volume in the dermal layer (3D)	Microscale	0.139
25	Average vessel area in the epidermal/SVP layer (2D)	Microscale	0.109
26	Area of the epidermal/SVP layer (2D)	Macroscale	0.128
27	Number of junction-to-endpoint branches in the dermal layer (3D)	Mesoscale	0.111
28	Thickness of the dermal layer (2D)	Macroscale	0.351
29	Thickness of the epidermal/SVP layer (2D)	Macroscale	0.082
30	Average vessel volume in the epidermal/SVP layer (3D)	Microscale	0.350
31	Vessel diameter in the dermal layer (3D)	Microscale	0.154
32	Vessel diameter in the epidermal/SVP layer (3D)	Microscale	0.274
All microscale features			0.485
All mesoscale features			***0.026**
All macroscale features			0.289

Supplementary Table 9 | Comparison of features selected by nested CV and LOSO RF.

Feature Description	Median importance over 30 trials	Order by nested CV	Order by LOSO RF
Number of vessels in the epidermal/SVP layer (2D)	0.049	1	1
Number of junction-to-junction branches in the epidermal/SVP layer (2D)	0.048	2	4
Number of junctions in the epidermal/SVP layer (2D)	0.047	3	2
Number of junction-to-junction branches in the dermal layer (2D)	0.045	4	3
Thickness of the dermal layer (3D)	0.041	5	5
Average vessel length of the dermal layer (3D)	0.039	6	–
Total area of vessels of the dermal layer (3D)	0.039	7	6
Average vessel length of the dermal layer (2D)	0.037	8	8
Thickness of the epidermal/SVP layer (3D)	0.035	9	12
Area of the dermal layer (2D)	0.035	10	7
Number of junction-to-endpoint branches in the epidermal/SVP layer (2D)	0.033	11	9
Area of the dermal layer (3D)	0.032	12	15
Total area of vessels in the epidermal/SVP layer (3D)	0.032	13	14
Number of junction-to-junction branches in the epidermal/SVP layer (3D)	0.031	14	18
Total area of vessels in the epidermal/SVP layer (2D)	0.030	15	22
Number of vessels in the dermal layer (3D)	0.029	16	13
Number of vessels in the epidermal/SVP layer (3D)	0.029	17	11
Number of junction-to-junction branches in the dermal layer (3D)	0.029	18	21
Number of junctions in the epidermal/SVP layer (3D)	0.029	19	16
Number of junction-to-endpoint branches in the epidermal/SVP layer (3D)	0.029	20	10
Average vessel volume in the epidermal/SVP layer (2D)	0.029	21	25
Area of the epidermal/SVP layer (3D)	0.029	22	23
Number of junctions in the dermal layer (3D)	0.029	23	20
Average vessel volume of the dermal layer (2D)	0.028	24	17
Average vessel volume of the dermal layer (3D)	0.028	25	24
Total area of vessels in the dermal layer (2D)	0.028	26	19
Area of the epidermal/SVP layer (2D)	0.026	27	26
Thickness of the epidermal/SVP layer (2D)	0.025	28	29
Thickness of the dermal layer (2D)	0.025	29	28
Average vessel volume of the epidermal/SVP layer (3D)	0.025	30	30
Number of junction-to-endpoint branches in the dermal layer (3D)	0.022	31	27
Number of vessels in the dermal layer (2D)	0.020	32	–
Average vessel diameter in the dermal layer (3D)	0.015	33	31
Number of junctions in the dermal layer (2D)	0.014	34	–
Average vessel diameter in the epidermal/SVP layer (3D)	0.007	35	–
Number of junction-to-endpoint branches in the dermal layer (2D)	0.001	36	–
Vascular length-to-width ratio in the dermal layer (2D)	0.000	37	–
Average vessel length in the epidermal/SVP layer (2D)	0.000	38	–
Average vessel diameter in the epidermal/SVP layer (2D)	0.000	39	–
