# Supplementary material for: Dermal features derived from optoacoustic tomograms via machine learning correlate microangiopathy phenotypes with diabetes stage
Source: Nat Biomed Eng. 2023 Dec 4;7(12):1667–82. doi: 10.1038/s41551-023-01151-w (PMC10727986; doi:10.1038/s41551-023-01151-w)
Supplement: Supplementary file 2 — Reporting Summary [file 41551_2023_1151_MOESM2_ESM.pdf]

nature portfolio

Corresponding author(s): Vasilis Ntziachristos

Last updated by author(s): Oct 23, 2023

Reporting Summary

Nature Portfolio wishes to improve the reproducibility of the work that we publish. This form provides structure for consistency and transparency in reporting. For further information on Nature Portfolio policies, see our Editorial Policies and the Editorial Policy Checklist.

Statistics

For all statistical analyses, confirm that the following items are present in the figure legend, table legend, main text, or Methods section.

n/a	Confirmed	
☐	☒	The exact sample size (n) for each experimental group/condition, given as a discrete number and unit of measurement
☐	☒	A statement on whether measurements were taken from distinct samples or whether the same sample was measured repeatedly
☐	☒	The statistical test(s) used AND whether they are one- or two-sided<br>*Only common tests should be described solely by name; describe more complex techniques in the Methods section.*
☐	☒	A description of all covariates tested
☐	☒	A description of any assumptions or corrections, such as tests of normality and adjustment for multiple comparisons
☐	☒	A full description of the statistical parameters including central tendency (e.g. means) or other basic estimates (e.g. regression coefficient) AND variation (e.g. standard deviation) or associated estimates of uncertainty (e.g. confidence intervals)
☐	☒	For null hypothesis testing, the test statistic (e.g. F
☒	☐	For Bayesian analysis, information on the choice of priors and Markov chain Monte Carlo settings
☐	☒	For hierarchical and complex designs, identification of the appropriate level for tests and full reporting of outcomes
☒	☐	Estimates of effect sizes (e.g. Cohen's d, Pearson's r), indicating how they were calculated

Our web collection on statistics for biologists contains articles on many of the points above.

Software and code

Policy information about availability of computer code

Data collection	No software was used for data collection.
Data analysis	The code for the data analysis is available on Zenodo at https://doi.org/10.5281/zenodo.10047634.

For manuscripts utilizing custom algorithms or software that are central to the research but not yet described in published literature, software must be made available to editors and reviewers. We strongly encourage code deposition in a community repository (e.g. GitHub). See the Nature Portfolio guidelines for submitting code & software for further information.

Data

Policy information about availability of data

All manuscripts must include a data availability statement. This statement should provide the following information, where applicable:

- Accession codes, unique identifiers, or web links for publicly available datasets
- A description of any restrictions on data availability
- For clinical datasets or third party data, please ensure that the statement adheres to our policy

The post-processed data (calculated features) for each individual are provided as Supplementary information. The trained models and the optoacoustic data are available for research purposes from the corresponding author on reasonable request.

Field-specific reporting

Please select the one below that is the best fit for your research. If you are not sure, read the appropriate sections before making your selection.

☒ Life sciences ☐ Behavioural & social sciences ☐ Ecological, evolutionary & environmental sciences

For a reference copy of the document with all sections, see nature.com/documents/nr-reporting-summary-flat.pdf

Life sciences study design

All studies must disclose on these points even when the disclosure is negative.

Sample size	No sample-size calculations were performed to determine sample sizes, because this pilot study was exploratory.
Data exclusions	Data were excluded because of quality reasons. Participants were excluded because of multiple comorbidities.
Replication	No measures for reproducibility were applied.
Randomization	The participants were allocated to the experimental groups according to their clinical picture (in particular, healthy volunteers, and patients with diabetes).
Blinding	Blinding was not relevant to the study, because the participants had to be chosen according to their clinical picture.

Reporting for specific materials, systems and methods

We require information from authors about some types of materials, experimental systems and methods used in many studies. Here, indicate whether each material, system or method listed is relevant to your study. If you are not sure if a list item applies to your research, read the appropriate section before selecting a response.

Materials & experimental systems

n/a	Involved in the study	
☒	☐	Antibodies
☒	☐	Eukaryotic cell lines
☒	☐	Palaeontology and archaeology
☒	☐	Animals and other organisms
☐	☒	Human research participants
☐	☒	Clinical data
☒	☐	Dual use research of concern

Methods

n/a	Involved in the study	
☒	☐	ChIP-seq
☒	☐	Flow cytometry
☒	☐	MRI-based neuroimaging

Human research participants

Policy information about studies involving human research participants

Population characteristics	In total, 115 individuals (40 healthy volunteers and 75 patients, clinically diagnosed with diabetes) were included in the study. The mean age of the healthy volunteers was 43.9 ± 16.11, and 15 males/25 females were recruited. The mean age of the patients with diabetes was 68.52 ± 14.95, and 49 males/26 females were recruited. A detailed description of the population demographics and clinical characteristics is provided in Supplementary Table 1.
Recruitment	The participants were recruited in the Diabeteszentrum am Marienplatz, Forschergruppe Diabetes e.V. am Helmholtz-Zentrum München and Klinikum rechts der Isar. They were placed in a dark and quiet room with normal temperature (about 23°C) and asked to lie in a supine position. Patients with systemic immunosuppressive therapies, UV therapy 4 weeks before the start of the study, in pregnancy or breast feeding period, or with psychiatric comorbidities, were excluded.
Ethics oversight	Ethics Committee of the Technical University of Munich.

Note that full information on the approval of the study protocol must also be provided in the manuscript.

Clinical data

Policy information about clinical studies

All manuscripts should comply with the ICMJE guidelines for publication of clinical research and a completed CONSORT checklist must be included with all submissions.

Clinical trial registration	Not applicable, as the study was not interventional.

Study protocol	Protocol #109/17 S, Protocol # 326/19 S, Protocol #323/21 S
Data collection	The participants were recruited in the Diabeteszentrum am Marienplatz, Forschergruppe Diabetes e.V. am Helmholtz-Zentrum München and Klinikum rechts der Isar. They were placed in a dark and quiet room at normal temperature (≈ 23°C), and asked to lie in a supine position. The recruitment took place from 2017 to 2019.
Outcomes	Not applicable, as the study was exploratory.
